# Enhancing communication with bereaved relatives about emergency and critical care trials (ENHANCE): a mixed-methods study

**DOI:** 10.1136/bmjopen-2025-106677

**Published:** 2026-02-26

**Authors:** Hannah Doughty, Elizabeth Deja, Bridget Young, Ingeborg Welters, Victoria Shepherd, Sarah Milosevic, Kellie O’Hara, Julie Carman, Vinoth Sankar, Emma Thomas-Jones, Joanne Euden, Kerry Woolfall

**Affiliations:** 1 Department of Public Health, Policy and Systems, University of Liverpool, Liverpool, UK; 2 Liverpool John Moores University, Liverpool, UK; 3 Department of Cardiovascular and Metabolic Medicine, University of Liverpool, Liverpool, UK; 4 University Hospitals of Liverpool Group, Liverpool, UK; 5 Liverpool Centre for Cardiovascular Science, Liverpool, UK; 6 Cardiff University, Cardiff, UK; 7 Centre for Trials Research, Cardiff University, Cardiff, UK; 8 ENHANCE PPI Representative, Sepsis Survivor and Volunteer for UK Sepsis Trust, Liverpool, UK

**Keywords:** Adult intensive & critical care, ACCIDENT & EMERGENCY MEDICINE, Clinical trials, Quality Improvement, Research Design, Health policy

## Abstract

**Abstract:**

**Objectives:**

Clinical research in emergency and critical care is vital, but recruitment and consent are complex. Research may be conducted without prior consent when patients are critically ill, and interventions are time critical. Some patients may die before research participation can be discussed with relatives, leaving the bereaved unaware of their involvement. This study explored potential communication strategies for informing bereaved relatives when a patient has died following enrolment into an emergency or critical care study without prior consent.

**Design and setting:**

A mixed-methods study using a telephone survey and semi-structured interviews conducted simultaneously. The survey was conducted within a National Health Service Trust in North West England with relatives of deceased study participants. Semi-structured interviews were conducted with bereaved relatives and research and clinical staff across the UK, and medical examiner (ME)/ME officers based in England and Wales. Quantitative data were analysed descriptively, and qualitative data were analysed using reflexive thematic analysis. Data were synthesised using a constant comparison approach.

**Participants:**

11 bereaved relatives completed the survey. 53 individuals (21 research and clinical staff, 18 relatives and 14 MEs/officers) participated in semi-structured interviews.

**Results:**

Although many trials do not include a process for notifying bereaved relatives about research participation, most relatives valued the opportunity to learn about their family member’s participation, emphasising the importance of transparency and trust. However, some raised concerns over the potential burden of automatic disclosure by the ME service. Offering bereaved relatives the option to receive sensitively worded information about research involvement at an appropriate time, soon after death, was recommended.

**Conclusion:**

Bereaved relatives should have the choice to be informed about research participation without prior consent. Our findings support the need for transparent and sensitive communication and will contribute to future guidance for the design and conduct of adult emergency and critical care studies.

**STRENGTHS AND LIMITATIONS OF THIS STUDY**

- This study used a mixed-methods design, combining a survey with semi-structured interviews for in-depth insight and data triangulation.
- Conducting interviews with bereaved relatives and a range of healthcare professionals ensured sample variance and insights from key stakeholders to identify appropriate and feasible recommendations for future trials.
- Interview and survey responses were limited to bereaved relatives and healthcare professionals from England, Scotland and Wales.
- Of the 21 critical care trials that were screened in England and Wales, only two included a process for notifying bereaved relatives about research involvement, therefore limiting the number of eligible trials for the survey.

## Introduction

 Clinical research in emergency and critical care is vital for advancing medical knowledge and improving treatment for critically ill patients.^1^ However, recruitment and consent in this context raise practical and ethical challenges.^2^ Approximately 90% of patients in intensive care units (ICUs) lacked decisional capacity to provide informed consent at the point of admission.^3^ Mortality rates are high among severely ill patients,^3^ and excluding the most critically ill from research introduces bias and diminishes the validity of findings.^4^ When interventions are not time critical, in patients who lack capacity, models of consent include approaching the patient’s relative as personal consultee, or where it is not possible to do so, involving an independent professional consultee to decide whether to enrol the patient in research.^5 6^ In emergency settings requiring immediate intervention, it is often not appropriate to obtain consent from those who lack the capacity to provide it. Alternative consent models can be used whereby individuals are entered into a study using ‘Research Without Prior Consent’ (RWPC), also known as ‘deferred consent’.^5 6^ Consent from the patient is then sought on their recovery.^5 6^ However, some patients may die before consent is obtained, leaving bereaved relatives unaware of their participation or the use of their data. In a lactate-guided resuscitation trial in patients with sepsis, 11% of patients died before investigators could obtain consent from the patient or their ‘surrogate decision-maker’.^6^ Despite high mortality rates in adult ICU, there is no legal obligation to discuss research participation with bereaved relatives. As a result, emergency or critical care studies may not include processes for notifying bereaved relatives about research involvement,^7^ and little is known about relatives’ preferences for receiving this information.^2 8^ Clinicians may struggle to know when, or whether, it is appropriate to discuss research involvement with grieving relatives.^2 7^ Previous research conducted in paediatric settings found that professionals were apprehensive about communicating RWPC to parents of critically ill children.^9^

In 2020/21, the National Health Service (NHS) piloted a statutory medical examiner (ME) system in England and Wales to independently scrutinise deaths.^10^ MEs are senior medical doctors in the NHS, supported by ME officers (MEOs), who aim to improve communication with bereaved relatives by discussing the cause of death and addressing concerns.^11^ From 9 September 2024, all deaths that are not investigated by a coroner will be reviewed by an ME.^10^ It may be possible for MEs/MEOs to inform bereaved relatives about research participation in these routine discussions. However, little is known about the appropriateness and feasibility of providing this information through ME services, or whether bereaved relatives would prefer alternative methods of communication.^2^

The aim of this study was to assess and improve communication strategies with bereaved relatives when a patient dies after enrolment in an emergency or critical care study without prior informed consent. The objectives were to explore bereaved relatives’ preferences regarding being informed about their family member’s research participation and receiving study findings, and to assess the feasibility and acceptability of integrating this communication into routine contact made by the ME office after a death.

### Study design

This was a triangulation mixed-methods study using the convergence model,^12^ with quantitative and qualitative data collected and analysed simultaneously.

## Methods

### Study setting

The MEO-led telephone survey took place in an NHS Trust in England with relatives of deceased study participants. Semi-structured interviews were conducted with bereaved relatives and research and clinical staff across the UK, and ME/MEOs were based in England and Wales.

### MEO-led telephone survey

#### Scoping of trials to participate in ENHANCE

A review of trial protocols was conducted by JE within trauma and emergency care and critical care specialities at three NHS Trusts (Liverpool Heart and Chest Hospital NHS Trust, The Walton Centre NHS Foundation Trust and Liverpool University Hospitals NHS Foundation Trust (LUHFT)), to identify potential interventional trials for inclusion in the MEO-led survey.

Inclusion criteria were trials: (1) involving participants aged 16+years; (2) where participants may have been recruited without prospective consent (either from the patient, their legal representative or consultee); and (3) where the protocol indicated bereaved relatives would be informed about their family member’s involvement. As the ME service was only introduced in England and Wales,^10^ only trials in these countries were eligible. Chief investigators and/or trial managers of eligible trials were contacted by KW and JE and invited to participate. Due to the sensitive nature of the survey, a pilot phase was conducted in one NHS Trust (LUHFT) with emergency and critical care specialities. On participation, the MEO reviewed Trust records to identify relatives of patients who had died during participation in those trials.

#### Development of the survey

The purpose of the MEO-led survey was to assess the effectiveness of an automatic notification system for informing bereaved relatives that their family member had participated in a research study that used RWPC, before their death. The survey aimed to assess awareness of research participation, communication preferences and whether the ME service was an appropriate method for delivering information about research participation. The MEO-led survey was developed from previous research^2 7^ and in collaboration with our enhancing communication with bereaved relatives about emergency and critical care trials (ENHANCE) study team and patient and public (PPI) representatives. The survey consisted of six researcher-derived questions relating to demographic information, relatives’ awareness of their family members’ involvement in research, their desire for further information about the research and their preferred communication methods (see box 1).

Box 1 **Medical examiner officer-led survey questions**

**Question**

1. Gender.
2. Ethnicity.
3. First part of your postcode.
4. Your relative (*name*) took part in a research study (*study name*) while they were recently being cared for in (*hospital*)*,* were you aware of that? (*Add very brief description of condition/treatment which was the focus*)
5. Would you like to receive some further information about their involvement in the (*study*)?
6. How would you like this information to be sent to you?

ENHANCE, enhancing communication with bereaved relatives about emergency and critical care trials.

#### Participant eligibility and recruitment

The survey was conducted between August 2023 and April 2024 (see ‘scoping of trials to participate in ENHANCE’ in the methods section, for trial eligibility criteria). We invited eligible research teams and MEs in the pilot Trust (based in North West England) to participate. Bereaved relatives were identified for the survey through: (1) participating trial research nurses notifying the ME office of a participant’s death within 24 hours using secure NHS email or (2) MEO searches of electronic records for the name of selected trials before contacting bereaved relatives, to identify if the deceased family member was a trial participant.

#### Procedure

The MEO used their expertise in communicating with recently bereaved relatives to determine if the survey should be broached during their call, refraining from doing so if they believed relatives were too distressed. The MEO administered the survey to relatives who provided consent and responses were recorded in the web-based platform Jisc,^13^ with access by the University of Liverpool research team. Contact details were collected for relatives wishing to discuss study participation with the relevant study team or to take part in an interview. If a relative expressed interest in discussing their deceased family member’s study participation, their contact details were securely provided to a knowledgeable research nurse, who contacted them via their preferred method. Since the MEO call occurs shortly after death, we initially aimed to recruit up to 10 relatives at one NHS Trust (LUHFT) to assess the potential burden. Based on this review, we planned to then review and consider opening the survey to up to two additional NHS Trusts under the sponsor organisation.

### Semi-structured Interviews

Topic guides were created based on previous literature^14^,^18^ and iteratively developed following each interview. Interviews with relatives explored perspectives on being informed about their family member’s participation in a study, preferred communication methods and any questions or concerns about receiving this information (see online supplemental resource 2). Interviews with ME/MEOs and clinical and research staff explored views and experiences of communicating study participation to bereaved relatives, perceptions of current methods and views on potential future approaches (see online supplemental resources 3 and 4).

#### Participant eligibility and recruitment

Semi-structured interviews were conducted between June 2023 and February 2024. For relatives of deceased participant interviews, individuals were eligible if they were: (1) relatives of deceased adult patients who had been enrolled in an emergency or critical care study in England and/or Wales where the patient died after enrolment; or (2) relatives who were suddenly bereaved, and/or relatives of patients who died in hospital following a critical or emergency illness in the last 3 years. For ME/MEO interviews, individuals were eligible if they were (1) ME/MEOs working in NHS Trusts or Health Boards and/or (2) clinical or research staff involved in recruiting to emergency and critical care studies in adults, working in NHS Trusts or Health Boards.

Participants were either recruited through purposive and snowball sampling, the MEO-led survey, social media advertisements or relevant support groups. Based on previous research^14^,^18^ and the need for sample variance, we aimed to sample approximately 20–30 bereaved relatives, 10–15 ME/MEOs and 10–15 research and clinical staff.

#### Procedure

HD (PhD, female, research associate) and ED (PhD, female, research fellow) managed interview recruitment and conducted all interviews for both relatives and research and clinical staff (see online supplemental resources 5 and 6). For relatives of deceased participants, following advice from our PPI partners, we delayed reaching out to survey participants for approximately 2–4 weeks after the MEO call, considering their recent bereavement. Due to the potential for distress, relatives in Merseyside were given the option of a remote or face-to-face interview, due to their proximity to the research base. Those living outside this area were provided with the option of a remote interview. Remote interviews were conducted either over the telephone or using the online videoconferencing platforms, Teams or Zoom. All professionals were provided with the option to participate in remote interviews. At the beginning of each interview, HD/ED explained the study, addressed questions and obtained informed consent. After the interviews, relatives received a £30 Amazon voucher to thank them for their time. Participants could also share information about the study with individuals who they thought might be eligible, helping to recruit additional participants through snowball sampling. The interview process was consistent across relatives, research/clinical staff and ME/MEOs. However, research/clinical staff and ME/MEOs were not provided with the option of a face-to-face interview or a participation voucher.

To ensure sample diversity, we recruited both relatives and healthcare staff based on several factors, including gender, geographical location, clinical or emergency care experience, professional role and prior involvement in emergency or critical care studies. Sample adequacy and data quality were continuously assessed during the interview process and we determined information power had been achieved when there was sample diversity, and breadth and depth to the data collected.^19^

### Patient and public involvement

We established a Study Management Group (SMG), including a critical illness survivor (JC) who was a coinvestigator and had extensive experience supporting bereaved relatives. JC was crucial to our study design and recruitment. JC ensured that all study-related materials were designed with sensitivity and that recruitment advertisements were appropriate to reach the intended audience. SMG meetings were held monthly via Microsoft Teams.

We also established a Study Advisory Group (SAG), which included a PPI partner (OJ) who is a lead support nurse for the UK charity, Sepsis Trust. OJ played a pivotal role in study design and recruitment, promoting the study through the Sepsis Trust and its networks. SAG meetings were held approximately every 6 months via Microsoft Teams. PPI partners provided study oversight and were involved in the interpretation and dissemination of study findings.

### Data analysis

Quantitative survey data were exported from Jisc^13^ into IBM SPSS Statistics for Windows, V.28 and analysed using descriptive statistics. Percentages were rounded to the nearest integer and may not total 100%. Qualitative data from semi-structured interviews were analysed using reflexive thematic analysis^20^ by HD, ED and KW (professor, female, social scientist) (see table 1). Interviews were audio recorded, transcribed *verbatim* by UK Transcription, checked for accuracy and anonymised by HD/ED. NVivo V.12 was used to organise data and facilitate analysis. Analysis was interpretive and iterative,^21 22^ and coding was conducted by HD and ED, with regular discussions with KW to support reflexivity and interpretation agreement. Qualitative and quantitative data were synthesised using constant comparison, informed by grounded theory.^23^,^1^ Allocated unique identification numbers and pseudonymised quotes are presented to accompany themes. Qualitative data are reported according to the Consolidated Criteria for Reporting Qualitative Research checklist (see online supplemental resource 7).^24^

**Table 1:** Approach to reflexive thematic qualitative data analysis

Stage	Description
1. Familiarising oneself with the data	HD and ED read and reread transcripts, noting down initial ideas on themes.
2. Generating initial codes	HD and ED began by systematically engaging with the data and generating initial data-driven codes and concepts, identifying meaning throughout the dataset. There was no limit on the number of codes created. Coding was iterative and interpretative and was conducted by HD and ED, with regular discussions with KW to support reflexivity and interpretation agreement.
3. Constructing themes and developing the coding framework	HD and ED reviewed codes to identify areas of similarity or overlap and grouped codes together to create broad themes. HD and ED gave themes meaning by going through the data associated with all similar codes, and although themes were linked to the aim and objectives of the study, they were not led by these.
4. Reviewing potential themes	HD refined, combined or discarded themes at this stage and incorporated data from across the dataset as a whole, not just as responses to specific questions.
5. Defining and naming themes	HD defined and named the themes establishing scope, focus and coherence. These were reviewed by ED and KW to support interpretation agreement.
6. Producing the report	HD wrote up the results and tested how well the themes worked in relation to the whole dataset. During this phase, data were collated to create an analytical narrative and presented with illustrative quotes. It was important to present themes in a logical order, to tell a story where each theme builds on or contradicts the previous theme. This step involved revisiting the research question, notes from the earlier stages of familiarisation and coding and ensuring the final themes answered the aim and objectives of this research. Final discussion and development of selected themes occurred during the write-up phase.

## Results

### Scoping of trials to participate in ENHANCE

JE identified 76 trials registered under the specialities of ‘Emergency and Trauma Care’ or ‘Critical Care’ across the three Liverpool NHS Trusts. Of those 76 trials, 21 were screened and identified as potentially eligible (eg, used RWPC and were still actively recruiting). Of those 21, only two trials met the inclusion criteria, 11 studies were either no longer recruiting or did not have an available protocol. Of the remaining eight studies, six involved RWPC, but only two mentioned informing relatives about participation if consent was not given before death. Both trials (PROcalcitonin and NEWS2 Evaluation for Timely Identification of Sepsis and Optimal Use of Antibiotics in the Emergency Department (PRONTO) and Intensive Care Unit Randomised Trial Comparing Two Approaches to Oxygen Therapy (UK-ROX)) agreed to participate. PRONTO compared procalcitonin-supported assessment with standard care for suspected sepsis in adults.^25^ UK-ROX investigated whether conservative oxygen therapy improves outcomes compared with usual oxygen therapy.^26^

## Participant characteristics

### MEO-led telephone survey demographics

11 bereaved relatives participated in the survey. Most were male (7/11; 64%), living in North West England (9/11; 82%) and self-identified as white British (8/11; 73%), white—Other (2/11; 18%) and white Irish (1/11; 9%). The MEO-led survey was not extended to additional NHS Trusts due to concerns raised in interviews about automatic notification (see findings below).

### Semi-structured interviews

82 potential bereaved relatives were identified as potential interview participants, with 6% identified from the survey and 94% from social media. On review, 54% of the social media responses were identified as imposters^27^ and excluded from follow-up. One interview was conducted where ED raised concerns regarding the authenticity of the participant. HD independently reviewed the transcript using guidance from literature on identifying imposter participants.^28 29^ After consultation with the SMG and SAG, the data for this participant were excluded from the analysis, resulting in 18 bereaved relative interviews.

**Figure 1:**
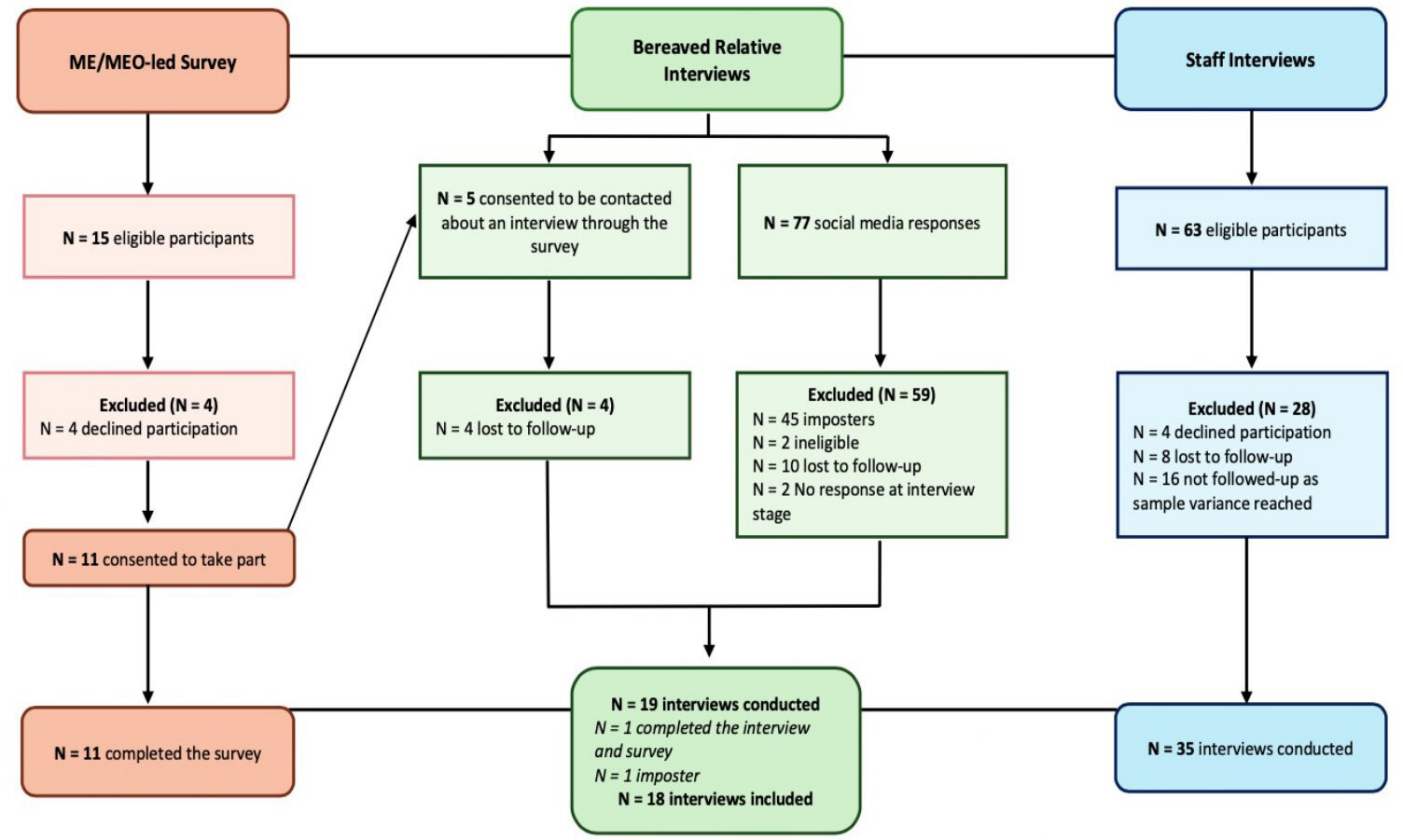
Recruitment figure. Figure 1 outlines the recruitment process, including the number of individuals screened, reasons for exclusion and the number of participants included in the MEO-led telephone survey (N = 11) and the semi-structured interviews (N=53). ME, medical examiner; MEO, medical examiner officer.

HD/ED conducted 18 bereaved relative interviews with 15 female and 3 male relatives; 6 were spouses, 9 were children and 3 were siblings of the deceased. Their ages ranged from 27 to 83 years (mean=52 years), and relatives were living in the UK (England (11/18; 61%), Wales (5/18; 28%), Scotland (2/18; 11%)). Of these relatives, 5/18 (28%) described that their family member had participated in research and 2/18 (11%) had provided consent on behalf of their family member in an emergency situation. One relative (1/18; 6%), identified through the MEO-led survey, had been notified of their family member’s involvement in a research study after their death. 35 interviews were conducted with 21 research and clinical staff (21/35; 60%) and 14/35 (40%) ME/MEOs. Professionals were based in England (32/35; 91%), Wales (2/35; 6%) and Scotland (1/35; 3%) and had varying levels of experience in their roles ranging from 6 months to 29 years. Collectively, they had experience of over 42 emergency or critical care studies. See figure 1 for recruitment figures and table 2 for demographic characteristics.

**Table 2:** Relatives’ demographic information (N=18)

Characteristics	Responses	N (%)
Gender	Female	15 (83)
	Male	3 (17)
Age	Range	27–83
	Median	52 years
Ethnicity	White—British	12 (67)
	White—Other	2 (11)
	Caucasian	2 (11)
	White—Welsh	1 (6)
	Missing data	1 (6)
Employment	Health or social care	6 (33)
	Business and other services	6 (33)
	Public sector or education	4 (22)
	Manufacturing, construction or agriculture	1 (6)
	Unemployed	1 (6)
Place of residence	England	11 (61)
	Wales	5 (28)
	Scotland	2 (11)
Relationship to the deceased	Child	9 (50)
	Spouse	6 (33)
	Sibling	3 (17)

All interviews were conducted remotely; 11/18 (61%) relative interviews were conducted via Teams/Zoom and 7/18 (39%) were conducted via telephone. The majority of staff interviews were conducted via Teams/Zoom (32/35; 91%) and 3/35 (9%) were conducted via telephone. All interviews ranged from 18 to 102 min (mean=44 min).

### Findings

Data synthesis led to four main themes including (1) ‘The potential burden of automatic disclosure of research participation’; (2) ‘The need for transparency to prevent a breakdown of trust’; (3) ‘Early integration and format of research participation communication’ and (4) ‘Who should be responsible for providing information about research participation?’.

#### The potential burden of automatic disclosure of research participation

Most bereaved relatives (8/11; 73%) who completed the survey were unaware their family member had participated in a research study whilst in hospital. Although personal communication with MEOs delivering the survey indicated relatives did not react negatively to this disclosure, some interview participants raised concerns that disclosing research participation without prior consent might cause unnecessary distress or *‘*put people over the edge’ (R10).

Some professionals expressed concern that relatives may react negatively to automatic disclosure. One doctor described an incident informing relatives about a clinical trial, which led to an inquest and a member of their research team taking prolonged leave.

It came about during the course of conversations that a patient had been enrolled in a trial… the family chose to divert all of their hostility and their frustration into the fact that the person had been in the trial. And the whole thing ended up with a very untidy inquest, and… we lost a very experienced member of our research nurse team as a result of it (HCP06, Doctor).

#### The need for transparency to prevent a breakdown of trust

When survey participants were asked if they would like more information about their family members’ research involvement, responses were mixed, with just over half (6/11; 55%) wishing to receive more information (see table 3). During interviews, most relatives expressed a preference for being given the opportunity to find out about their family member’s research involvement. They described how bereaved relatives may experience comfort from knowing about their family member’s research participation and use of their data to help others.

**Table 3:** MEO-led telephone survey results (n=11)

Survey item	N (%)
1. Gender	
Male	7 (64)
Female	4 (36)
2. Ethnicity	
White British	8 (73)
White Irish	1 (9)
White—Other	2 (18)
3. Location of residence	
Liverpool	7 (64)
St Helens	1 (9)
Reading	1 (9)
Wirral	1 (9)
Missing data	1 (9)
4. Your relative (name) took part in a (name) research study while they were recently being cared for at (hospital), were you aware of that?	
Yes	1 (9)
No	8 (73)
Unsure	1 (9)
Missing data	1 (9)
5. Would you like to receive some further information about your relative’s involvement in the (study name)?	
Yes	6 (55)
No	4 (45)
6. How would you like this information to be sent to you?	
Email	4 (36)
Telephone	1 (9)
Post	1 (9)
Missing data	5 (45)

MEO, medical examiner officer.

Because you would think that something positive had come out of it. Something that might help in the future (R06).

Relatives and health professionals emphasised the importance of transparency to prevent suspicion that clinicians were *‘*hiding information’ (HCP04, ME) as ‘otherwise it could be misconstrued’ (R02). It was also described that *‘*anything that compromises that [trust], should be discouraged’ (HCP04, ME) and *‘*there has to be a defendable reason why you wouldn’t inform people’ (HCP06, Doctor).

Participants stated that bereaved relatives might learn about research participation through different channels, such as requests to access their family member’s medical records. This could potentially undermine trust if they had not been informed beforehand.

There’s always, at the back of my mind, that relatives can request their deceased relatives’ notes. And how would they feel if, for some reason, they decided to… apply to access their notes, read it, and thought, ‘Why did they not tell me?’…It raises the trust issue again (HCP 18, Doctor).

#### Early integration and format of research participation communication

The majority of relatives interviewed stated they would prefer to receive information about research participation early in the grieving period, with preferences ranging from 1 to 7 days following death. One relative stated that receiving this information *‘*sooner’ would be *‘*better’, as they would prefer to *‘*deal with it at [the] moment in time when it’s raw’ (R08). Another relative described that if they were informed about research participation months later, it could *‘*open that wound wider again’ (R04). This sentiment was echoed by professionals, with one describing it as important *‘*to make that contact’ and not to *‘*let too much time pass’ (HCP18, ME). However, another professional highlighted the complexity of bereavement, describing that a *‘*one-size-fits-all solution’ may not be appropriate (HCP27, ME).

Relatives stated they would have appreciated the opportunity to have a choice over their preferred method of contact if they did wish to know about their family member’s participation in research. Most relatives said they would prefer to receive this information in written format, rather than via telephone. Receiving an unexpected phone call was seen as potentially distressing as *‘*there is that emotional shock element [if someone calls] you out of the blue at any time’ (R01). This finding was consistent with survey results. Among relatives who indicated a contact preference (6/11; 55%), the majority (4/6; 67%) said they would prefer to receive this information via email, while only (1/6; 17%) wished to receive this via telephone, or letter (1/6; 17%).

#### Who should be responsible for providing information about research participation?

During the interviews, various options were explored as to which professional(s) should be responsible for delivering information about a patient’s research participation, if this was requested by relatives. Most interviewees did not express a preference. Communication from either the clinical, research or bereavement teams was viewed as acceptable, provided the information was given in a timely manner. Many relatives emphasised the importance of ensuring that the person delivering the information about research participation is equipped to answer questions relatives might have as they would not want to receive *‘*half information’ (R02).

There were mixed views regarding whether it would be appropriate to embed research communication within the ME service. Some relatives felt that the ME/MEO call could serve as an opportunity for early contact following a death, avoiding the need for additional contact.

I think if you’re going to get that call anyway… I think that is a good time to then just share that information (R01).

However, not all agreed. Most professionals, including ME/MEOs, raised concerns about this method, describing how the ME/MEO role should serve as an independent process and it should not be an *‘*additional role’ for them to *‘*inform the family members’ (HCP33, ME).

## Discussion

Bereaved relatives expressed mixed views about being informed of a family member’s research participation without prior consent before death. Most relatives expressed a preference for being informed and receiving study findings, emphasising the importance of transparency in building trust. However, some raised concerns about the potential burden of automatic disclosure. Offering relatives the option to learn whether their family member participated in research may balance transparency, whilst not causing burden for those who do not wish to know about such participation. An example phrasing for this communication could be *‘*this is a research active hospital, and some patients may have been involved in research during their care, before they passed away. Would you like me to check if your family member was involved in research during their hospital admission?*’* Communication should occur promptly after death (eg, within a week) via the relative’s preferred contact method and be delivered by a staff member able to address questions, such as someone from the clinical, research or bereavement team.

Our initial review of study protocols to inform the MEO-led survey found that most of the sample of critical care studies did not include a process for notifying bereaved families about emergency and critical care research participation.^7^ This may stem from concerns among clinicians, researchers and ethicists about increasing relatives’ emotional burden.^2 7 30^ However, most bereaved relatives wanted to be informed about their family member’s participation, suggesting that the current practice of not communicating research involvement after death does not meet the wishes of the bereaved. This finding is consistent with studies conducted in paediatric emergency research, which reported initial anxiety among professionals about communicating RWPC to parents of critically ill children.^9^ However, parental responses were generally positive, particularly when the reasons for not obtaining prior consent and the nature of the interventions were explained clearly.^2 18 31^ Many parents expressed hope that their child’s participation could help others in the future.^18 31^

Relatives’ have the right to request their family members’ medical records^32^ and could find out about research participation through such access.^33^ Our study highlighted that if bereaved relatives discovered research participation unexpectedly through reading their family member’s medical records, this could lead to distress and concerns about mistrust around the cause of death. Healthcare professionals have a duty of candour—an ethical responsibility to be open about care, especially when a patient has died.^34^ Some professionals described how providing relatives with the option to receive this information could help to prevent unexpected discoveries. While the role of the ME service is to improve communication with bereaved relatives,^11^ our study found that ME staff believed communicating about research involvement through the ME service would be inappropriate and should remain independent from research. Relatives wanted a choice to receive this information and expressed a preference for written communication within a week of their family member’s death, including contact details of relevant staff so they could ask questions. Relatives highlighted the importance of clear communication when informing bereaved relatives about research conducted without prior consent. This finding aligns with previous research, which emphasises the essential role of effective communication by professionals when interacting with grieving families.^35^

Our study was the first to explore whether bereaved relatives would wish to be informed about their adult family member’s participation in RWPC, as well as their preferences for the timing and method of communication. Our findings have informed recommendations for ensuring transparent, sensitive and timely communication, which have been used to inform guidance (ENHANCE | Institute of Population Health | University of Liverpool) for best practices in the design and conduct of adult emergency and critical care studies. The use of quantitative and qualitative methods and analysis enabled data triangulation, enhancing breadth and depth of insight, and facilitated a comprehensive understanding of the research topic. Interviewing bereaved relatives with varied relationships to the deceased and healthcare professionals with bereavement communication and research roles ensured we included a broad range of perspectives. Of the 21 critical care trials screened in England and Wales, only two included a process for notifying bereaved families. This significantly limited the number of trials eligible for inclusion in the survey. However, 11 of the 15 (73%) eligible relatives identified took part. Our findings are limited to the views of the bereaved and NHS staff from England, Scotland and Wales. Relatives who agreed to participate in our study may have been more likely to hold positive views about research, and our findings may not represent the views of those who declined to participate.

Our findings have important implications for the design and conduct of future emergency and critical care research. Currently, most research protocols lack a clear pathway for informing bereaved relatives about their family member’s research participation prior to death. This does not align with many bereaved relatives’ preferences, or the professional duty of candour. Research teams should incorporate transparent and timely communication options in their protocols. Our findings suggest that most bereaved relatives would appreciate the opportunity to learn about their family member’s participation in research; however, not all wish to be informed. It is crucial to offer relatives the choice of whether to receive this information, ensuring their preferences are respected while maintaining honesty and transparency.

## Supplementary material

10.1136/bmjopen-2025-106677 online supplemental file 1

## Data Availability

No data are available.
