# Supplementary material for: Enhancing communication with bereaved relatives about emergency and critical care trials (ENHANCE): a mixed-methods study
Source: BMJ Open. 2026 Feb 26;16(2):e106677. doi: 10.1136/bmjopen-2025-106677 (PMC12959003; doi:10.1136/bmjopen-2025-106677)
Supplement: online supplemental file 1 [file bmjopen-16-2-s001.docx]

**Online Resource 1.** ENHANCE Protocol


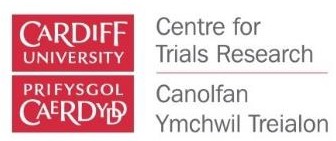

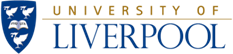

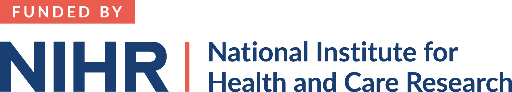


**Enhancing communication with bereaved relatives about emergency and critical care trials**

STUDY SHORT TITLE

**ENHANCE**

**Version 1.2, 20.10.23**

This study was funded by the National Institute for Health and care Research (NIHR) Research for Patient Benefit (RfPB) programme (project number: 201067). The views and opinions expressed therein are those of the authors and do not necessarily reflect those of the RfPB Programme, NIHR, NHS or the Department of Health.

##### **Signature page**

| The undersigned confirm that the following protocol has been agreed and accepted and that the Chief Investigator agrees to conduct the trial in compliance with the approved protocol and will adhere to appropriate research governance framework and any subsequent amendments of regulations, Good Clinical Practice (GCP) guidelines, the Sponsor’s Standard Operating Procedures (SOPs) and other regulatory requirements as amended.  I agree to ensure that the confidential information contained in this document will not be used for any other purpose other than the evaluation or conduct of the clinical investigation without the prior written consent of the Sponsor.  I also confirm that I will make the findings of the trial publicly available through publication or other dissemination tools without any unnecessary delay and that an honest, accurate and transparent account of the trial will be given; and that any discrepancies from the trial as planned in this protocol will be explained.  **For and on behalf of the Sponsor:** | |
| --- | --- |
| Signature: | Date: |
| Name (please print): |  |
| Position: |  |
| **Chief Investigator:**  **Signature: 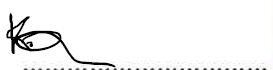** **Date 20.12.22**  **Name: Dr Kerry Woolfall**  **Position: Reader in Health Research Methodology** | |

**Research reference numbers**

**Protocol version number and date**

Version 1.2 October 2023

**IRAS Number:** 324927

**REC Reference**

**Sponsor name and reference: The University of Liverpool reference: UoL001761**

**Funder name and reference**

National Institute for Health and care Research (NIHR), Research for Patient Benefit reference: 204067

**Chief Investigator and co-lead**

Dr Kerry Woolfall and Dr Joanne Euden

**Sponsor representative Mrs Karen Jennings Wilding**

**ENHANCE study contacts**

| **Chief Investigator** | Dr Kerry Woolfall  Reader in Health Research Methodology University of Liverpool  Department of Public Health, Policy and Systems Institute of Population Health and Society,  Room G09, Whelan Building, Liverpool L69 3GL  Email: [k.woolfall@liverpool.ac.uk](mailto:k.woolfall@liverpool.ac.uk) Tel: 07872963676 |
| --- | --- |
| **Co-Lead** | Dr Joanne Euden  Centre for Trials Research  College of Biomedical and Life Sciences Cardiff University  7th Floor, Neuadd Meirionnydd Heath Park  Cardiff CF144YS  Email: [eudenj@cardiff.ac.uk](mailto:eudenj@cardiff.ac.uk) Tel: 02922 510771 |
| **Sponsor** | The University of Liverpool is the research Sponsor for this Study. It is recognised that as an employee of the University the Chief Investigator has been delegated specific duties, as detailed in the Sponsorship Approval letter.  For further information regarding the sponsorship conditions, please contact:  Mrs. Karen Jennings-Wilding  Senior Clinical Research Governance Manager  [Sponsor@liverpool.ac.uk](mailto:Sponsor@liverpool.ac.uk) |
| **Funder** | National Institute for Health and care Research, Research for Patient Benefit |

| **Name** | **Institution** | **Role/expertise** |
| --- | --- | --- |
| Kerry Woolfall | University of Liverpool | Chief investigator – Research methodology, emergency and critical care trial recruitment  and consent |
| Joanne Euden | Cardiff University | Co- Chief Investigator –  Management of emergency and critical care trials |
| Bridget Young | University of Liverpool | Co-applicant - qualitative and patient centred research, communication and  recruitment to clinical trials |
| Julie Carman | Patient Partner | Co-applicant, sepsis survivor who provides support for bereaved families as part of  UK Sepsis Trust |
| Ingeborg Welters | University of Liverpool | Co-applicant - intensive care  consultant and trialist |
| Victoria Shepherd | Cardiff University | Co-applicant – Qualitative researcher, trials involving  patients who lack capacity |
| Sarah Milosevic | Cardiff University | Co-applicant - Researcher with qualitative expertise in  clinical trials |
| Karen Poole | Liverpool University Hospitals NHS Foundation  Trust | Co-applicant - Medical Examiner |
| Elizabeth Deja | University of Liverpool | Co-applicant - Qualitative researcher, time critical  clinical trials |
| Vinoth Sanker | Liverpool University Hospitals NHS Foundation  Trust | Co-applicant -Medical Examiner |
| Emma Thomas-Jones | Cardiff University | Co-applicant – trialist |

**Study Management Group**

**Contents**

[Protocol summary 6](#_bookmark0)

1. [Background 7](#_bookmark1)
2. [Aims and Objectives 8](#_bookmark2)
3. [Study design and setting 8](#_bookmark3)
   1. [Study flowchart 8](#_bookmark4)
   2. [Work package 1- Survey of bereaved families 9](#_bookmark5)

[Patient identification 10](#_bookmark6)

[Survey recruitment and consent 10](#_bookmark7)

- 1. [Work package 2- interviews with families and healthcare professionals 10](#_bookmark8)

[Recruitment of bereaved relatives 11](#_bookmark9)

[Recruitment of Medical Examiners and research staff 12](#_bookmark10)

- 1. [Work package 3: Guidance/toolkit development 13](#_bookmark11)

[Recruitment 13](#_bookmark12)

1. [Dissemination and outputs 14](#_bookmark13)
2. [Project management 14](#_bookmark14)
3. [Patient and Public Involvement 15](#_bookmark15)
4. [Regulatory issues 15](#_bookmark16)
   1. [Ethics approval 15](#_bookmark17)
   2. [Confidentiality 15](#_bookmark18)
   3. [Data storage and use of personal data 15](#_bookmark19)
   4. [Study management 16](#_bookmark20)
   5. [Sponsorship and indemnity 16](#_bookmark21)
   6. [Audits 16](#_bookmark22)
   7. [Archiving 16](#_bookmark23)
   8. [Funding 16](#_bookmark24)

[9. References 17](#_bookmark25)

[Appendix 1 – Amendment History 17](#_bookmark26)

#

# **Protocol summary**

| **Title:** | Enhancing Communication with Bereaved Relatives About Emergency and Critical Care Trials |
| --- | --- |
| **Short Title/acronym** | ENHANCE |
| **IRAS number** | 324927 |
| **REC number** | 23/YH/0052 |
| **Sponsor name & reference** | University of Liverpool |
| **Funder name & reference** | NIHR RfPB |
| **Design** | Mixed methods study |
| **Overall aim** | To assess and improve communication strategies with bereaved relatives when a patient has died following enrolment into an emergency/critical care trial without prior informed consent. |
| **Anticipated study duration** | 16 months |

# **Background**

Clinical research in emergency or critical care settings is vital, yet the process of recruitment and consent is practically and ethically complex, particularly as only around 10% of ICU patients have capacity to provide informed consent for research and there is a high risk of mortality amongst severely ill patients (1). Excluding the most critically ill patients from a clinical trial creates bias and reduces the validity and relevance of the research. In emergency research, where participants are unable to provide consent, alternate models of consent are often used. These include approaching the patient’s relatives to seek their advice or consent or, where it is not practicable to do so, involving an independent professional, such as a doctor in deciding to enroll the patient in research (2, 3). Consent is then sought from patients when they recover. However, patients enrolled in emergency research based on consent or agreement from a healthcare professional may die before the study is discussed with relatives, leading to situations where bereaved families are unaware that their family member has participated in a clinical trial and that their data will be used for research. Participants' dying prior to obtaining consent from the patient or their relative is not uncommon in emergency trials, for example in a trial of lactate-guided resuscitation of patients with sepsis, 11% patients died prior to the investigators obtaining consent from the patient or their 'surrogate decision-maker' (3). There is a lack of knowledge about whether relatives wish to be informed of participation in research, and if so, when and how they would prefer to be informed (4).

Doctors can struggle to know when, or whether it is appropriate to discuss a deceased patient’s research involvement with grieving family members. In 2020/21 a new NHS system, called the medical examiner system, began. Medical examiners are specially trained doctors and nurses who contact relatives shortly after a patient has passed away. The medical examiner aims to help improve communication with bereaved families and provide information about the cause of death. It may be possible for medical examiners to inform bereaved relatives about research participation into their discussions. However, there is a need to seek relatives, medical examiners and researcher views on whether this is possible or appropriate. There is also a need explore whether relatives want to be informed at all, and potential alternative options of informing them about research participation and the best timing and way to do this.

Uncertainty about the most appropriate approach to communication with bereaved relatives about trial participation, and worry about causing additional burden for grieving families, is an ongoing concern for clinicians, researchers, ethicists and patient partners involved in the design and conduct of clinical trials in these challenging settings (5).

# **Aims and Objectives**

The aim of the study is to assess and improve communication strategies with bereaved relatives when a patient has died following enrolment into an emergency/critical care trial without prior informed consent. The specific objectives are to:

- Objective 1: Exploring the preferences of bereaved relatives about being informed of the patient’s involvement in the trial, to identify for example, their views on the timing and management of the disclosure, staff involved and whether relatives would wish to receive the findings of the trial once these are available.
- Objective 2: Assess the feasibility and acceptability of embedding additional communication about trial participation during the routine process of the Medical Examiner (ME) Office contacting relatives about a death.
- Objective 3: Use findings to develop a framework/guidance tool to aid clinicians, ME Offices and trial teams on how best to communicate with bereaved relatives in future emergency and critical care research as well as within the ME system

# **Study design and setting**

Mixed methods study involving relatives of deceased trial participants, ME Office and research staff involved in emergency and critical care trials in England and Wales.

### **Study flowchart**


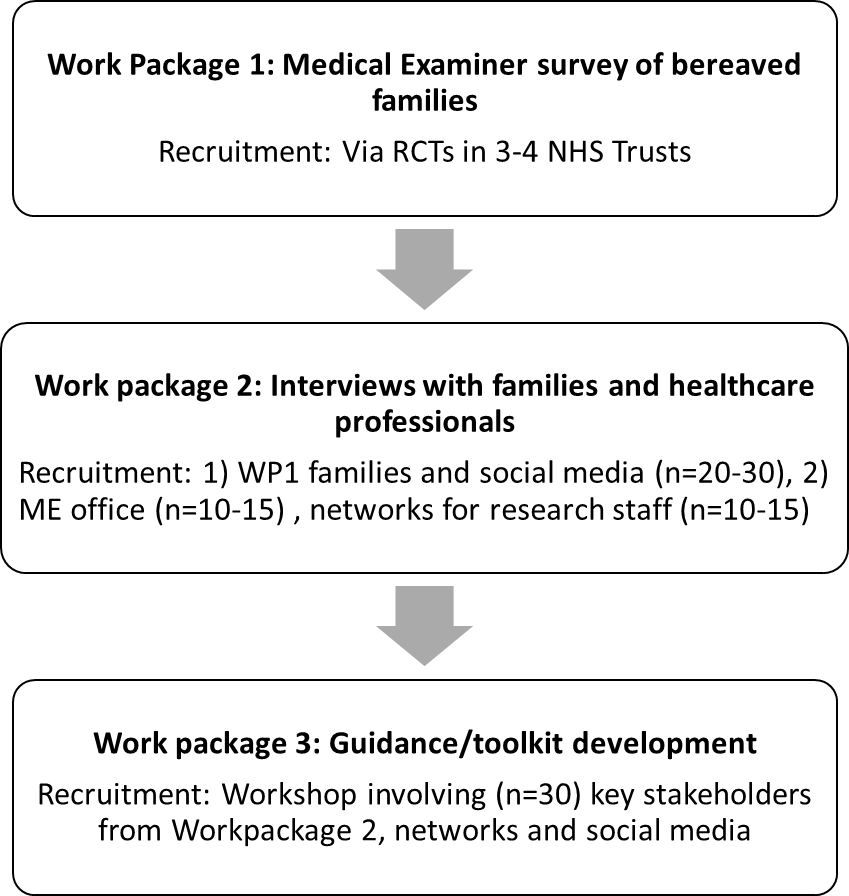
The study will comprise three phases over a 16-month time frame (shown in the study flowchart below).

## **Work package 1 - Survey of bereaved families**

To address study objective 1, this work package will involve a survey of families and/or next of Kin of patients who have died after taking part in a clinical trial without prior consent. The questions will assess relatives’ awareness of the patient’s involvement in a clinical trial, whether the family would like further information about the trial their relative was involved in and/ or to be contacted by a member of the trial team, and their preferences for contact (format and timing).

**Target population:** Bereaved families of patients recruited to research in 3-4 NHS Trusts

**Method:** ME Office and bereavement nurses at 3-4 NHS Trusts will embed a brief set of questions into their calls with families after the death of patient enrolled in a trial (see study inclusion criteria below).

- - 1. Interventional trials with 3-4 NHS Trusts in England and/or Wales;
    2. The trial involved research participants aged 18 +;
    3. where participants may be recruited without prospective consent (either from the patient or their legal representative or advice from a consultee) and;
    4. where the protocol indicates bereaved relatives will be informed about the patient’s

involvement.

####

#### **Patient identification**

There will be two approaches to the identification of patients available to participating trials/ trusts. These will be discussed at site set up to determine which approach fits best with the trials/trusts systems.

*Patient Identification approach 1*: Participating trial research nurses will identify a patient death and notify ME Office using secure NHS email within 24 hours of death (as ME calls with relatives take place within 24-48 hours of a patient death).

*Patient Identification approach 2*: Before ME Office contacts a bereaved relative they will search their electronic records for the name of a participating trial (e.g. PRONTO) to identify if the bereaved patient was a participant and whether they would be eligible for the survey (e.g. recruited without prior consent).

#### **Survey recruitment and consent**

ME Office will seek verbal consent from relatives for participation (WP1 survey questions and/or register interest in a WP2 interview- see below) towards the end of their call with families. As ME Office contact families within 24-48 hours of a death, families may be distressed or struggle to process much information. The medical examiner will use their extensive expertise in communicating with recently bereaved families to establish whether or not to conduct the questionnaire. If there are signs that the family are too distressed to invite them to participate in the brief questionnaire, they will not recruit them to the study.

**Conduct:** If consent is provided, ME Office will administer the questions, recording responses electronically (e.g. Qualtrics online). The questionnaire has intentionally been kept very brief and should only take 2-3 minutes to complete.

**Sample size:** As this study is purely a descriptive analysis no formal power calculation is needed, However, we will aim for a sample size based on cumulative potential mortality rates for our identified eligible trials using a 30% approached-to-consent rate. Families will be recruited to the survey using a sampling framework to help ensure equality, inclusion and diversity (e.g. range of trials, gender, and geographical/social economic diversity)

**Analysis: Quantitative** data from this survey will be exported from the online survey to SPSS. Data will be analysed descriptively, as is the intention of this survey. Open text responses will be analysed using a thematic (6) to identify patterns.

### **Work package 2- interviews with families and healthcare professionals**

**Method:** semi structured interviews conducted either face to face, online or via telephone

*Participant Inclusion criteria:*

- - 1. Relatives of adult patients enrolled in an Emergency or Critical Care trial in England and/or

Wales where the patient died after enrolment;

2) Relatives who are suddenly bereaved, and/or relatives of patients who have died in hospital

following a critical or emergency illness in the last three years

3) Medical Examiners and Medical Examiner Officers working in NHS Trusts or Health Boards;

4) Research staff (doctors/nurses) working in NHS Trusts or Health Boards involved in recruitment

to emergency and critical care trials involving adults.

#### **Recruitment of bereaved relatives**

Recruitment route 1

Through Work package 1, at the end of the questionnaire ME Office will invite relatives to state whether or not they would like to be sent information about participation in an interview. The contact details of those who register interest will be forwarded to the study qualitative researcher. Two forms of contact will be sought (e.g. email and telephone) alongside their preferences about the preferred mode of contact.

Recruitment route 2

To assist sample diversity and recruitment opportunities, we will use social media advertising to recruit bereaved families who have had an adult relative die following an admission to a UK emergency or critical care department in the last three years. This will include families who may, or may not know, if their relative took part in a trial when they were in hospital. Adverts will be tailored to purposive sample for inclusivity and diversity (e.g. to include families from both England and Wales and variance in socioeconomic and ethnic background). We will also seek to include a range of close relatives (both in terms of relationship, individual characteristics, trials involved etc). We anticipate these relatives will mainly be partners, husbands, wives and children, but will purposively sample to also include other relatives, such as aunts, uncles and grandparents where possible.

The advert will include contact details for the researcher who will then check eligibility against the inclusion criteria.

**Arranging interviews:** Upon confirmation of eligibility and screening for diversity, the researcher will send a study participant information leaflet and consent form to relatives and arrange a convenient time and format (telephone or online) for interview. For recruitment route 1: We will contact relatives recruited approximately 2-4 weeks after consent to contact. The option of face-to-face interviews will be provided to those who live in the Merseyside area. The information leaflet will outline the study aims, what would be involved, any potential risks and benefits associated with participation and research team contact details.

**Informed consent:** The researcher will begin telephone or online interviews by explaining the aims of the study, providing an opportunity for questions and verbally obtaining informed consent for the study. This will involve the researcher reading each aspect of the ENHANCE Participant Consent Form to participants, including consent for audio recording and to receive a copy of the findings when the study is complete. The researcher will tick each box on the consent form when the participant provides verbal consent and then sign the consent form. Informed consent discussions will be audio recorded for auditing purposes. Written informed consent will be sought from participants who are interviewed face to face. A copy of the consent form will be sent to the participant after the interview and the original will be kept at UoL.

**Conduct:** Interviews will explore topics such as how relatives felt/or would feel about being informed about their family member’s inclusion in a trial (either by the trial team as part of trial processes or ME in Phase 1 of this study), whether they wanted/or would want to be told, and how they would prefer to receive this information and any questions or concerns. Interviews with families recruited via social media will involve an initial description of the ME service and study aims, before exploring their views on the areas outlined above. Throughout this research we will ensure all contact with families is appropriate and sensitive to the distressing nature of this topic. We will provide families with details of support services (e.g. CRUSE, ICU Steps) in the participant information sheet who families can contact following the interview if they wish. After the interview the ENHANCE researcher will send relatives a £30 Amazon voucher to thank them for their time.

#### **Recruitment of Medical Examiners and research staff**

We will recruit medical examiners and research staff involved in UK adult emergency and critical care trials with a range of experiences and expertise (including MO/MEO, bereavement nurses) and geographical location. ME Office and research staff will be sent email invitations to participate using our contacts and national networks (e.g. Trial Managers Network). Social media recruitment will also be used aid purposive sampling to ensure inclusivity and diversity and inclusion of those beyond the group involved in work package 1.

**Arranging interviews:** Upon confirmation of eligibility, the researcher will send a study participant information leaflet and consent form to relatives and arrange a convenient time and format (telephone or online) for interview. The information leaflet will outline the study aims, what would be involved, any potential risks and benefits associated with participation and research team contact details.

**Informed consent:** the same informed consent process outlined above for relatives will be used for medical examiners and research staff interviews.

**Conduct:** ME Office and research staff interviews will explore their views and experiences on: communicating trial participation with bereaved relatives; current approaches used by trials in these settings; the feasibility and acceptability of communicating such information in future trials; and most appropriate study output (e.g. guidance or toolkit). Interview topic guides will be developed iteratively, with previously unanticipated topics added and discussed with participants as interviews and analyses progresses (e.g. early findings from relative interviews may be fed into research staff and ME Office interviews). This approach is important to ensure the expressed needs of bereaved relatives are explored in-depth and to identify common views to inform the development of draft guidance or toolkit (Work package 3).

**Sample size**: Recruitment will stop when it is judged that data collected has sufficient ‘information power’ (7). To establish whether the point of information power has been met we will consider the extent to which data addresses: the study aims; sample specificity (e.g. participants’ experience

relevant to the study aims and sample diversity); our reflexive approach to theory and analysis (8, 9); and the quality of interview dialogue (i.e. the extent to which it provides an in-depth insight into participant views and experiences(7). Based on previous research and the need for sample variance, we anticipate interviewing 20-30 bereaved relatives, 10-15 MEOs and 10-15 Research staff.

**Analysis:** Our approach to analysis will be interpretive, using a reflective thematic analysis approach for the analysis of interview data (8). We will synthesise WP1 and WP2 findings using the constant comparison approach(10), whilst drawing on foundational ethical principles and the research ethics literature to develop recommendations on enhancing communication with bereaved relatives

in future emergency and critical care trials as well as within the ME service. This will include an assessment and draft recommendations about the feasibility of embedding communication about trial participation during the future routine process of the ME Office contacting relatives about a death. Draft recommendations will be used to develop guidance or a toolkit (format informed by interview findings) for review in Work package 3.

### **Work package 3: Guidance/toolkit development**

We will conduct a workshop, inviting key stakeholders (involved and not involved in phase 1 and 2) to review and develop draft recommendations for a framework/guidance tool to aid clinicians, ME Office and trial teams on how best to support bereaved relatives in future emergency and critical care research and within the ME system.

The workshop will have a dual focus. It will firstly engage key stakeholders to help raise awareness of the study and forthcoming outputs as part of the dissemination strategy. Secondly, the workshop will bring together key stakeholders and PPI to develop the draft guideline or toolkit.

#### **Recruitment**

We aim to recruit approximately 30 participants to the workshop with a balance of groups (ME office/relatives/research staff/and HRA/REC representatives) whilst ensuring sufficient numbers of relatives are invited so there is a balance of public and professional attendees in each of the smaller groups on the day (see below). A matrix of key stakeholder groups will be developed to inform purposive sampling

We will send an email to phase 2 participants (if consent has been provided), as well as invitations through our networks, relevant charities, and social media advertising.

**Conduct:** The researcher will send a draft copy of the guidance document or toolkit to attendees to read before the workshop. The workshop will be held online to assist accessibility, with break out rooms used by facilitators (the ENHANCE researcher and co-applicants with qualitative expertise e.g. KW, ED, VS, BY) to go through the guidance document line by line in smaller groups. The workshop will begin with a welcome address and presentation of the study findings, guidance/toolkit development process, and agenda for the day. The attendees will then be invited to join breakout rooms with 5-6 attendees in each group with a mix of stakeholders including a balance of public and professionals. In each group a draft recommendations/toolkit area will be introduced by the facilitator and delegates. They will then be asked: ‘Do you have any initial comments or concerns about this recommendation/content? Three key areas will be explored including:

- Relevance of the draft recommendations/content: What is the current practice and would this change approaches? If so, how? Is the recommendation/content appropriate for professionals and relatives? Is there evidence, from practice or other sources, that has not been considered in developing these recommendations?
- Usefulness: How might these recommendations/content build on or change current approaches? What are the implications of this? Are there any ethical concerns that have not been addressed? Are the guidelines accessible, clear and workable in practice? Are they appropriate to different patient/relative groups?
- Feasibility: What are the barriers to/opportunities for implementation? What further resources, training or support might be needed to implement them? Attendees will be asked to identify the possible barriers or facilitators to successfully implementing the suggested approach/activity and potential solutions.

**Analysis:** All discussions will be digitally recorded and transcribed. QSR NVivo software will be used to assist in the organisation and indexing of qualitative data. Whilst analysis will be informed by the constant comparison approach of grounded theory (15), the focus will be modified to fit with the criterion of catalytic validity, whereby findings should be relevant to future research and practice. Workshop findings will be integrated into the development of final guidelines or toolkit, whichever study participants indicated would be the most useful output.

### **Dissemination and outputs**

To facilitate immediate patient benefit though the inclusion of the developed guidance/toolkit in emergency and critical care trial protocols and staff training, we will publish in open access peer reviewed journals and present at national and international scientific meetings, research training events, site initiation visits and send direct emails to critical care networks and trial PIs. We will work with our PPI partners to co-produce a lay summary of findings for dissemination through bereavement support groups and social media.

***End of study***

The End point of the study will when all data are analysed and study outputs are complete.

### **Project management**

This study involves a number of study team members from across the UK. To ensure effective team communication KW and JE will arrange regular (bi-monthly) online meetings to discuss project milestones, progress and issues arising. A shared folder in Microsoft teams will contain all up to date study documents for access by team members. These mechanisms will ensure effective communication within the team.

### **Patient and Public Involvement**

Our study team includes PPI partner (JC) who is a former critically ill patient who works with bereaved families. JC, alongside a wider and diverse group of PPI partners and key stakeholders (e.g. trialists, ethicists, ICU nurses) will form a study advisory group. A key purpose of the advisory group will be to involve families with lived experience of bereavement in each step of the study; from study and information material design, recruitment and conduct, interpretation of findings for guidance development and dissemination. We will work together with the advisory group to co- design lay summaries of the study results for disseminating our findings to for participants and to help us communicate the study results to a wider public audience. Advisory group members (bereaved relatives) will be offered appropriate training material to support them in their role and funded for the time involved in undertaking training. Members of our team (ED, KW, JC) are experienced in working with and supporting patient partners. Free training modules are accessible through NIHR Learning for Involvement and Imperial College London, including how to provide feedback on research documents.

1. **Regulatory issues**

### **Ethics approval**

The study will be conducted in accordance with the ethical principles originating in the Declaration of Helsinki and those in Good Clinical Practice. As we will interview parents (as well as NHS staff) NHS approval review will be required from the HRA. This will be sought through proportionate review by KW and JE.

### **Confidentiality**

The Chief Investigator will preserve the confidentiality of participants taking part in the study and will abide by the Data Protection Act 2018 and the UK GDPR as amended from time to time and any successor legislation in the UK and any other directly applicable regulation relating to data protection and privacy.

Data will be pseudo- anonymised. The University of Liverpool will keep identifiable information for 10 years after the study has finished. Data will be kept in a secure encrypted file on a University of Liverpool computer drive. Only the University of Liverpool research team will have access to the data for quality control, audit and analysis.

The University of Liverpool is the sponsor for this study and will act as the Data Controller for this study. This means that we are responsible for looking after your information and using it appropriately. Dr Kerry Woolfall, University of Liverpool acts as the Data Processor for this study.

### **Data storage and use of personal data**

Contact details will be sought by the researcher to arrange interviews, conduct telephone interviews and send copies of the consent form and study findings (if consented for). Consent will be sought for those who wish be contacted about future research. Personal contact details will not be used for any other purpose.

All personal data will be held at the University of Liverpool. No personal data will be transferred electronically. All data will be securely stored in a locket cabinet or in an encrypted electronic file. All original files will be labelled with a unique identity number, encrypted and held on password protected University of Liverpool desktop computers. As soon as the digital recordings have been transcribed, the digital files will be archived securely at the University of Liverpool and retained for up to 10 years for auditing purposes. Audio recording is necessary to ensure full and accurate accounts of interviews.

Recorded consent discussions will not be transcribed but labelled with a unique identifying number and stored securely in an encrypted file on a secure University of Liverpool drive for auditing purposes. These digital files will be retained for up to 10 years for auditing purposes.

Publication of direct quotations from participants is necessary to report the results of qualitative research, but no identifying information will appear in transcripts and, therefore, in the quotations. Consent will be sought for audio recordings and secure upload of the recordings to the UK Transcription website for professional transcription (in accordance with the Data Protection Act 2018).

### **Study management**

All day-to-day management of the ENHANCE Study will be the responsibility of the SMG. Members of the SMG will include the Chief Investigator, Co- lead and the co-investigators. The SMG will meet regularly to discuss management and progress of the ENHANCE study.

### **Sponsorship and indemnity**

#####

##### **Sponsor Details**

| Sponsor Name: | University of Liverpool |
| --- | --- |
| Address: | Clinical Directorate |
| Contact: | Sponsorship team |
| Email: | [Sponsor@liverpool.ac.uk](mailto:Sponsor@liverpool.ac.uk) |

The University of Liverpool holds Indemnity and insurance cover with Newline Insurance Company, which apply to this study.

### **Audits**

The study may be subject to inspection and audit by the University of Liverpool under their remit as sponsor and other regulatory bodies to ensure adherence to GCP and the UK Policy Framework for Health and Social Care Research (v3.2 10th October 2017).

### **Archiving**

Data and all appropriate documentation should be stored for a minimum of 10 years after the completion of the study.

### **Funding**

The ENHANCE study is supported by grant funding from the NIHR RfPB programme (reference: 204067) The SMG will review financial aspects of the study and report to sponsor.

### **References**

- 1. Harvey SE, Elbourne D, Ashcroft J, Jones CM, Rowan KM. Informed consent in clinical trials in critical care: experience from the PAC-Man Study. Intensive Care Med 2006;2020–2025
  2. Fox EE, Bulger EM, Dickerson AS, del Junco DJ, Klotz P, Podbielski J, et al. Waiver of consent in noninterventional, observational emergency research: the PROMMTT experience. J Trauma Acute Care Surg. 2013;75(1 Suppl 1):S3-8.
  3. Jansen TC, Bakker J, Kompanje EJ. Inability to obtain deferred consent due to early death in emergency research: effect on validity of clinical trial results. Intensive Care Med. 2010.
  4. Fitzpatrick A, Wood F, Shepherd V. Trials using deferred consent in the emergency setting: a

systematic review and narrative synthesis of stakeholders’ attitudes. Trials. 2022;23(1):411.

- 1. Paddock K, Woolfall K, Frith L, Watkins M, Gamble C, Welters I, et al. Strategies to enhance recruitment and consent to intensive care studies: a qualitative study with researchers and patient– public involvement contributors. BMJ Open. 2021;11(9):e048193.
  2. Braun V, Clarke V, Hayfield N, Terry G. Thematic Analysis. In: Liamputtong P, editor. Handbook of Research Methods in Health Social Sciences. Singapore: Springer Singapore; 2019. p. 843-60.
  3. Malterud K, Siersma VD, Guassora AD. Sample Size in Qualitative Interview Studies: Guided by Information Power. Qual Health Res. 2016;26(13):1753-60.
  4. Braun V, Clarke V. Reflecting on reflexive thematic analysis. Qualitative Research in Sport, Exercise and Health. 2019;11(4):589-97.
  5. Angen MJ. Evaluating Interpretive Inquiry: Reviewing the Validity Debate and Opening the Dialogue. Qualitative Health Research. 2000;10(3):378-95.
  6. Glaser B. The Constant Comparative Method of Qualitative Analysis. Social Problems. 1965;12(4):436-45.

#### **Appendix 1 – Amendment History**

| **Amendment No.** | **Protocol version no.** | **Date issued** | **Author(s) of changes** | **Details of changes made** |
| --- | --- | --- | --- | --- |
|  | V1.2 | 20.10.23 | Ms Hannah Doughty/Dr Beth Deja/Dr Kerry Woolfall | Relatives who are suddenly bereaved, and/or relatives of patients who have died in hospital following a critical or emergency illness in the last three years |

**Online Resource 2.** Bereaved Relatives Interview Schedule

**ENHANCE Interview Topic Guide (Relatives)**


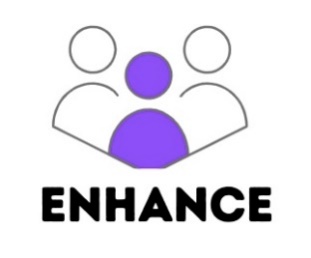


Please note: *Italic text indicates instruction for researcher and will not be read to participant*

Intro: My name is [*researcher name*] and I am a researcher from the University of Liverpool. Many thanks for agreeing to take part in the ENHANCE study.

(If online or telephone). Before we begin the interview, I need to obtain your consent for the study is that ok? (*Refer to instructions (in box) on the Participant Consent form including consent for audio recording of this discussion).*

**I will start with some questions about you if that’s ok**

**If RECRUITED THROUGH ME OFFICE: I will ask you about your experience of being invited to take part in the ENHANCE Study and then ask you some questions about how best to approach bereaved relatives about their family members involvement in a clinical trial. This work will help inform how we communicate with bereaved families in the future.**

**IF RECRUITED THROUGH SOCIAL MEDIA: I will then ask you some questions about how best to approach bereaved relatives about their family members involvement in a clinical trial. This work will help inform how we communicate with bereaved families in the future.**

**ALL If there is anything that you find difficult to talk about, please don’t feel that you have to, or if you want to stop the interview at any point, then please let me know. If there are any questions that you would rather not answer please say so and we can move on to the next. You can stop or pause the interview at any point.**

Before we start do you have any questions?

**About you**

**1. Please could you tell me your:**

- What year were you born?
- What is your occupation?
- What is the first part of your postcode?
- How would you describe your ethnic background?

**About your relative**

**2. I have a few questions about your relative *(Prompt: state relationship if known)*. I am aware this may be difficult so please take your time. Also, let me know if you would like to skip any of these questions**.

- Could you please tell me which hospital was your relative in when they passed away?
- How were you related? (*if not already known*)
- Were they in hospital for long? (*explore how long before they passed away*)
- What was the condition that caused their death?
- How old were they?
- Did you know if they were asked to take part in any research studies when they were in hospital?

**IF NO, go to question 3**

**IF YES**

- Could you please tell me what you can remember about the research studies? (Prompts: can you remember what the study/ies was/were called, or what the research was looking at? (*Go through each one in turn if multiple studie*s)
- Was your *{ADD RELATIONSHIP}* asked to take part in this study or were you asked to provide consent for their involvement in the study?

*If consented on their behalf:* Could you tell me about the conversations you had with staff about the study/studies before *{ADD RELATIONSHIP}* passed away.

- Can you recall who approached you about the study (Prompt: role rather than person)
- How did they approach you *(face-to-face, telephone)*?
- How did you feel about the research team approaching you about research at that point in time?
- Could the research team have improved how they approached you (*Prompt: better timing? Another type of contact such as phone call or face to face?)*
- How did you feel about making this decision?
- Did you speak to any other members of the family when making this decision?
- How long did you take to make the decision?
- What sort of things did you consider when making this decision?
- Did you consent to your *{ADD RELATIONSHIP}* involvement in the study (if approached about multiple studies go through each in turn)

**3) As discussed earlier, the ENHANCE study is looking at approaches to communicating with relatives when a patient has died after being enrolled in a clinical trial in an emergency situation**.

I want to explain this in a bit more detail if that’s ok and then ask you a few questions.

The life-threatening situation means that there isn’t time to speak to families about the patient’s involvement in the research, or seek their consent, as the medical team are focussed on caring for the patient. In these circumstances, patients are automatically included in the research. This is called research without prior consent, or deferred consent. All studies procedures are reviewed by a research ethics committee as well as other groups, such as the institution running the study and the study team, which includes patient representatives.

- What do you think about RWPC in emergency situations? (explore any concerns or potential benefits)

There are many different types of research studies. Some involve evaluating medications or new medical devises. Others might collect data without changing treatment in anyway. Previous research has shown that people’s views on being informed about trial involvement may or may not change based on the type of study. I am now going to explain two examples of studies carried out in an emergency setting that used RWPC and ask your views on whether or not you would have wishes to be informed about your relatives involvement in these studies.

The REMAP-CAP Trial looked at the treatment of COVID acquired pneumonia in intensive care units during the pandemic. The study aimed to find out the best combination of antibiotic, antiviral and steroids, to increase survival and improve health outcomes. The aim of this study was to keep track of what medication people were on to see who had the best outcomes.

Would you want to have been informed about your *{ADD RELATIONSHIP}* involvement in this clinical trial after they had passed away?

Would you have had any questions about their involvement in this trial?

Would you have had any concerns about their involvement in this trial without prior consent?

The AIRWAYS-2 Trial looked at the best way to keep a person’s airway open during a cardiac arrest.

It has been thought to be placing a breathing tube in the windpipe (tracheal intubation). However, attempting to place a breathing tube can cause complications such as accidental placement in the oesophagus (food pipe) rather than the airway, tissue damage and interruptions in chest compressions. It is possible that a newer method of airway management, inserting a supraglottic airway device, is better than tracheal intubation during cardiac arrest. Supraglottic airway devices are already used during routine anaesthesia in hospital; in emergency care, they are quicker to insert and cause less interruption to chest compressions.

Would you want to have been informed about your *{ADD RELATIONSHIP}* involvement in this clinical trial after they had passed away?

Would you have had any questions about their involvement in this trial?

Would you have had any concerns about their involvement in this trial without prior consent?

**4) Who do you think is the best person to contact bereaved relatives about their relatives’ enrolment in research? (explore if research team or doctor or nurse not involved in research)**

**ME OFFICE**

**5) In 2020/21, a new Medical Examiner system was rolled out in NHS Trusts and Health Boards across England and Wales. The Medical Examiner (ME), and associated Medical Examiner’s Officer (MEO), role aims to provide information about the cause of death to bereaved relatives and provide them with the opportunity to raise any concerns about the care their family member received, improve the overall quality of information collected and reported in relation to deaths in the UK.**

**(For ME recruits. ‘As you know’) Medical examiners currently contact families via telephone to discuss their relative’s death. The medical examiners are doctors with specialist bereavement training and bereavement nurses.**

- How would you feel if the medical examiner informed you about your *{ADD RELATIONSHIP}* involvement in research as part of the information they provide? Would you have any concerns? What questions would you want to ask?
- Of the three options we have discussed: 1) contact by research team, 2) contact by clinical team not involved in research and 3) contact by ME Office as part of their call, which would you prefer? Explore reasons

**6) If your *{ADD RELATIONSHIP}* had been involved in a trial, would you be interested in receiving the findings of the research when they became available?** IF yes, explore preferred format (e.g. via post, being provided with link to website and approximate date information will be posted, and alternatives?)

Opinions differ about whether or not to inform bereaved relatives of their family member’s enrolment in research. Some research teams do inform families after the patient has passed away. Other research teams don't do this. We're really interested in your views.

**7) Would you want to have been informed about your *{ADD RELATIONSHIP}* involvement in a clinical trial after they had passed away?**

(If no, explore reasons. Go to question 4)

If yes,

- When do you think would be the best time to contact bereaved families to inform them about their relatives’ involvement in research?
- How do you prefer to be contacted by the research team? *(prompt: for example, should this be a face-to-face discussion or another form of contact?)*
- How would you feel if you were informed via a letter? *(prompt- explore if letter should offer face to face discussion – what else should be included in the letter?)*
- How would you feel if you were informed by telephone?
- What is the key information you want to know about the research your relative was part of?

**8) Is there anything else that you think researchers need to consider when deciding when and how to inform relatives about research involvement after they have passed away?**

**Thank them for their time and highlight support services in information sheet.**

**Online Resource 3.** Medical Examiner (ME)/Medical Examiner Officer (MEO) Interview Schedule

**ENHANCE Interview Topic Guide (ME/MEO)**


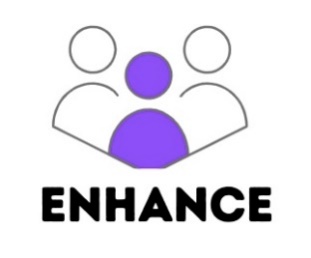


Please note: *Italic text indicates instruction for researcher and will not be read to participant*

Intro: My name is [*researcher name*] and I am a researcher from the University of Liverpool. Many thanks for agreeing to take part in the ENHANCE study.

(If online or telephone). Before we begin the interview, I need to obtain your consent for the study is that ok? (*Refer to instructions (in box) on the Participant Consent form including consent for audio recording of this discussion).*

**I will start with some questions about you**

**I will then ask you some questions about how best to approach bereaved relatives about their family members involvement in a clinical trial. This work will help inform how we communicate with bereaved families in the future.**

**ALL If there is anything that you find difficult to talk about, please don’t feel that you have to, or if you want to stop the interview at any point, then please let me know. If there are any questions that you would rather not answer please say so and we can move on to the next. You can stop or pause the interview at any point.**

Before we start do you have any questions?

**About you**

**1. Please could you tell me:**

- What is your occupation? (explore ME role and other clinical roles)
- How long have you been a ME?
- Which hospital do you work in?
- How would you describe your ethnic background?

**As described in the participant information leaflet, the ENHANCE study is looking at approaches to communicating with relatives when a patient has died after being enrolled in a clinical trial in an emergency situation**.

I will explain this in a bit more detail and then ask you a few questions.

The life-threatening situation means that there isn’t time to speak to families about the patient’s involvement in the research, or seek their consent, as the medical team are focussed on caring for the patient. In these circumstances, patients are automatically included in the research. This is called research without prior consent, or deferred consent. All study procedures are reviewed by a research ethics committee as well as other groups, such as the institution running the study and the study team, which includes patient partners.

**2) What do you think about RWPC in emergency situations? (explore any concerns or potential benefits)**

Opinions differ about whether or not to inform bereaved relatives of their family member’s enrolment in research. Some research teams do inform families after the patient has passed away. Other research teams don't do this. We're really interested in your views.

**3) Thinking about emergency or critical care research studies that are conducted without prior informed consent, do you think bereaved families should be informed about their relatives’ involvement in a study before they passed away?**

(If no, explore reasons. Go to question 4)

If yes, explore reasons and ask:

- When do you think would be the best time to contact bereaved families to inform them about their relatives’ involvement in research?
- How do you think they should be informed? (explore options such as face to face discussion, personalised letter, telephone) (go to question 4)

**There are many different types of research studies. Some involve evaluating medications or new medical devises. Others might collect data without changing treatment in anyway. Previous research has shown that people’s views on being informed about trial involvement may or may not change based on the type of study. I am now going to explain two examples of studies carried out in an emergency setting that used RWPC and ask your views on whether or not you think relatives should be informed their involvement in these studies.**

The REMAP-CAP Trial looked at the treatment of COVID acquired pneumonia in intensive care units during the pandemic. The study aimed to find out the best combination of antibiotic, antiviral and steroids, to increase survival and improve health outcomes. The aim of this study was to keep track of what medication people were on to see who had the best outcomes.

**Do you think relatives should be informed about involvement in this clinical trial after they had passed away?**

The AIRWAYS-2 Trial looked at the best way to keep a person’s airway open during a cardiac arrest.

It has been thought to be placing a breathing tube in the windpipe (tracheal intubation). However, attempting to place a breathing tube can cause complications such as accidental placement in the oesophagus (food pipe) rather than the airway, tissue damage and interruptions in chest compressions. It is possible that a newer method of airway management, inserting a supraglottic airway device, is better than tracheal intubation during cardiac arrest. Supraglottic airway devices are already used during routine anaesthesia in hospital; in emergency care, they are quicker to insert and cause less interruption to chest compressions.

**Do you think relatives should be informed about involvement in this clinical trial after they had passed away?**

**4) Have you been involved in informing bereaved families about their relatives’ involvement in a trial as part of the ENHANCE study?**

**Yes**

- When did you begin recruitment to the survey? (Prompt: confirm how many months that has been)
- In this time frame, approximately how many family members did you decide it would not be appropriate to broach the ENHANCE study? Could you tell me a bit more about how you make that decision not to broach the study survey?
- How many bereaved family members have you recruited to the ENHANCE survey?
- How many have said yes to taking part in the survey?
- How have bereaved family members reacted when you have mentioned research participation? (explore positive and negative responses)
- Do you think it is appropriate for medical examiners to inform bereaved family members about their relatives’ involvement in a study during their call? (explore responses)
- Based on your experience of broaching research involvement to relatives in ENHANCE are there any recommendations you would make if this approach was used in the future?

**No**

One potential way of informing bereaved families about research involvement is during your call as a medical examiner.

- What do you think about medical examiners informing bereaved family members about their relatives’ involvement in a study during their call (only where no consent has been obtained)? (explore responses including potential benefits, concerns)
- How would you feel if you were asked to broach the topic of research involvement before death with relatives during your call?
- Would there be any practical challenges if this conversation took place during your call? (for example, are you able to search for the names of trials in your records to identify whether a patient took part before they died? Explore other ways of identification in their trust)

**5) An alternative option is for the research team or clinical care team to contact bereaved families to inform them of research participation. What are your thoughts on this approach?** (explore both research team and clinical team contact).

- When do you think would be an appropriate time for them to contact families? (if they do think they should contact explore how)
- Of the options we have discussed: 1) contact by research team, 2) contact by clinical team not involved in research) 3) contact by ME Office as part of their call, which do you think is more appropriate? Explore reasons

**6) Is there anything else that you think we need to consider when making recommendations about when and how to inform relatives about research involvement after they have passed away?**

**7) As part of the study we plan to write a guidance document or toolkit to assist researcher and potentially medical examiners on how best to communicate with bereaved families about research participation following death. What type of document would you find most useful? (explore content, format (online or paper).**

**8) Do you have any additional comments before we finish today?**

**Thank them for their time.**

**Online Resource 4.** Research/ Clinical Staff Interview Schedule

**ENHANCE Interview Topic Guide (Research/Clinical Staff)**


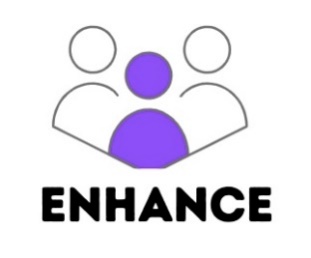


Please note: *Italic text indicates instruction for researcher and will not be read to participant*

Intro: My name is [*researcher name*] and I am a researcher from the University of Liverpool. Many thanks for agreeing to take part in the ENHANCE study.

If online or telephone. Before we begin the interview, I need to obtain your consent for the study is that ok? (*Refer to instructions (in box) on the Participant Consent form including consent for audio recording of this discussion).*

**I will start with some questions about you**

**I will then ask you some questions about the studies you have been involved in and approaches to communicating with bereaved relatives about their family members involvement in a clinical trial. This work will help inform how we communicate with bereaved families in the future.**

**ALL If there is anything that you find difficult to talk about, please don’t feel that you have to, or if you want to stop the interview at any point, then please let me know. If there are any questions that you would rather not answer please say so and we can move on to the next. You can stop or pause the interview at any point.**

Before we start do you have any questions?

**About you**

**1. Please could you tell me:**

- What is your occupation? (explore research and clinical roles)
- How long have you been involved in recruiting to research?
- How long have you been involved in emergency or critical care research?
- Please tell me the names of the ICU or ED clinical trials that you have been involved in recruiting to?
- Did any of these trials state in the protocol that relatives should be informed about a patients’ involvement in a trial when consent was not sought before they died?

If no or unsure, go to question 3

If yes, go through each trial in turn and ask:

- For the X trial, what did this aim to explore?
- What trial design was it (e.g. RCT, cluster RCT, platform)
- What approaches to consent were used in this trial (explore if informed consent/RWPC/consent from relative or doctor (professional or personal consultee/opt out)?
- When a patient died before consent was sought from either the patient or a family member, could you tell me how families were contacted? (explore methods in the protocol such as face-to-face, letter, telephone)
- Were you involved in any of these discussions? (If no, who was? If yes, explore how researcher felt about contacting families and how families responded to being informed that their relative had taken part in a trial)

**3) As described in the participant information leaflet, the ENHANCE study is looking at approaches to communicating with relatives when a patient has died after being enrolled in a clinical trial in an emergency situation**. **We are specifically looking at circumstances when patients are included in the research prior to seeking consent due to the time critical situation. So, research without prior consent, or deferred consent.**

- What is your experience of RWPC?
- What do you think about RWPC in emergency situations? (explore any concerns or potential benefits) (prompt about COVID study discussed prior to interview that resulted in family member complaints).
- Have you been involved in any emergency or critical care studies where there have been concerns about the use of RWPC? (explore if research was conducted without consent or if any approach for consent was taken by the team)
- Do you have any concerns about delaying treatment if consent is sought from consultee/professional or relative, rather than RWPC?

Opinions differ about whether or not to inform bereaved relatives of their family member’s enrolment in research. As you may know from first-hand experience, some research teams do inform families after the patient has died. Other research teams don't do this. We're really interested in your views.

**4) Thinking about emergency or critical care research studies that are conducted without prior informed consent, do you think bereaved families should be informed about their relatives’ involvement in a study in general?**

(If no, explore reasons. Go question 5)

If yes,

- Why do you think bereaved families should be informed about their relatives involvement in a study?

- When do you think would be the best time to contact bereaved families to inform them about their relatives’ involvement in research?
- How do you think they should be informed and by whom? (explore options such as face to face discussion, personalised letter, telephone) (go to question 5)

**5) One potential way of informing bereaved families about research involvement is during a call with a medical examiner, which usually takes place a day or two after death.**

What do you think about medical examiners informing bereaved family members about their relatives’ involvement in a study during their call (only where no consent was obtained when they were alive)? (Explore responses including any potential benefits, concerns)

**6) An alternative option is for the research team or clinical care team to contact bereaved families to inform them of research participation. What are your thoughts on this approach? (explore both research team and clinical team contact).**

- If the research team/clinical team was responsible for informing bereaved families about their relatives research participation, how would you feel about delivering this information? Explore concerns/benefits
- When do you think is the appropriate timing for the research/clinical team to contact families? (if they do think they should contact explore how)
- Of the options we have discussed: 1) contact by research team, 2) contact by clinical team not involved in research) 3) contact by ME Office as part of their call, which do you think is more appropriate? Explore reasons

**7) Is there anything else that you think we need to consider when making recommendations about when and how to inform relatives about research involvement after they have passed away?**

**8) As part of the study we plan to write a guidance document or toolkit to assist researcher and potentially medical examiners on how best to communicate with bereaved families about research participation following death. What type of guidance would you find most useful? (explore content, format (online or paper). If we find from this study that relatives do want to be informed about their relatives involvement after they have died, in a study using RWPC, would you use this guidance in practice?**

**9) Do you have any additional comments before we finish today?**

**Thank them for their time.**

**
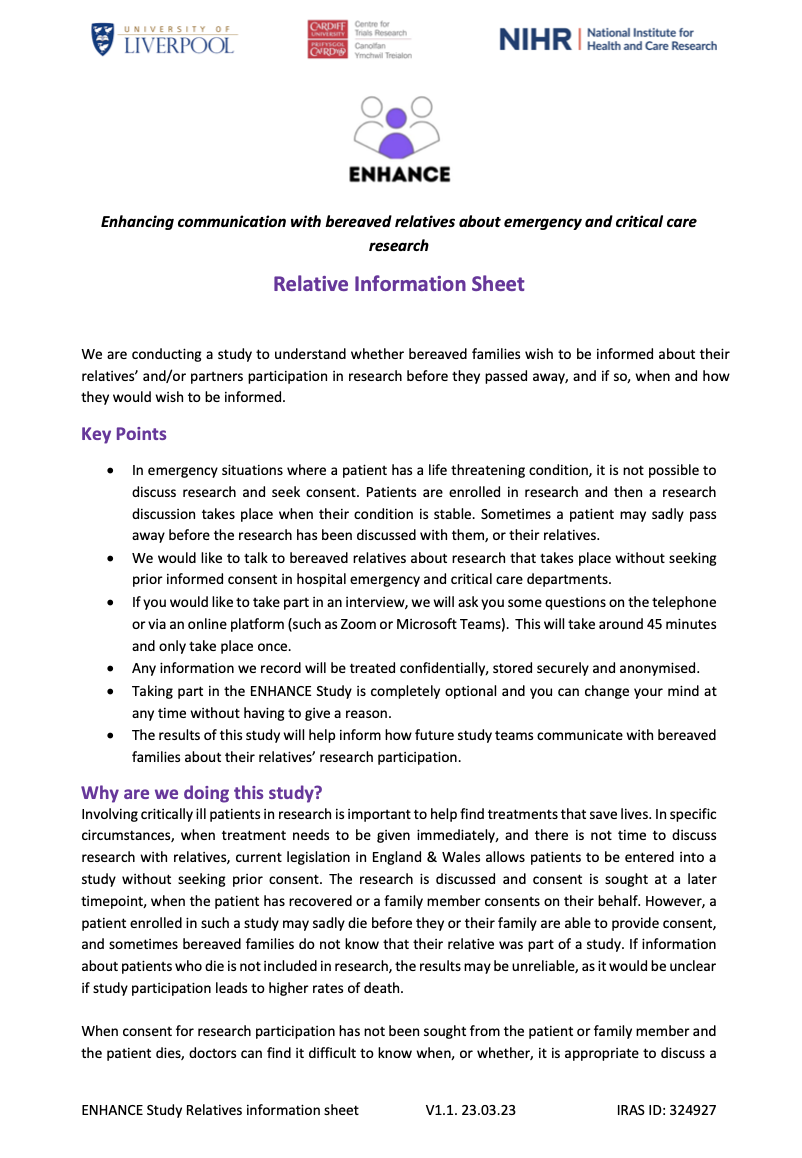
Online Resource 5.** Participant Information Sheet (Relatives)

**
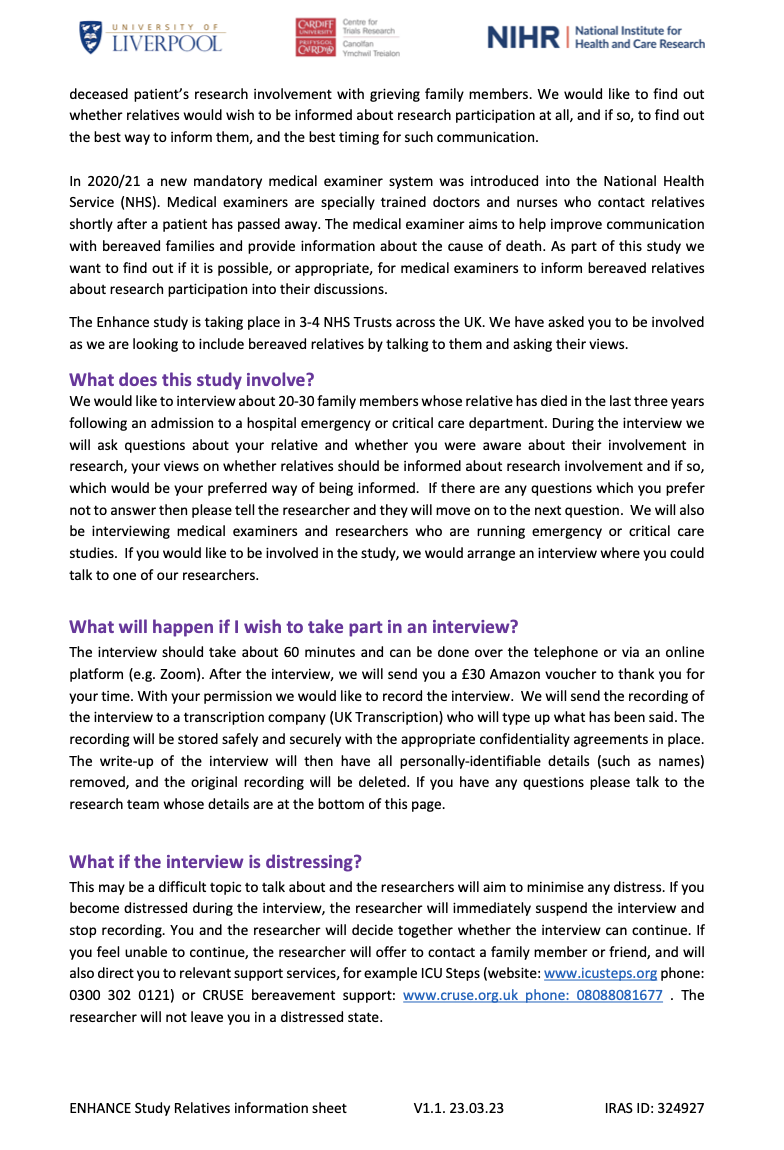
**

**
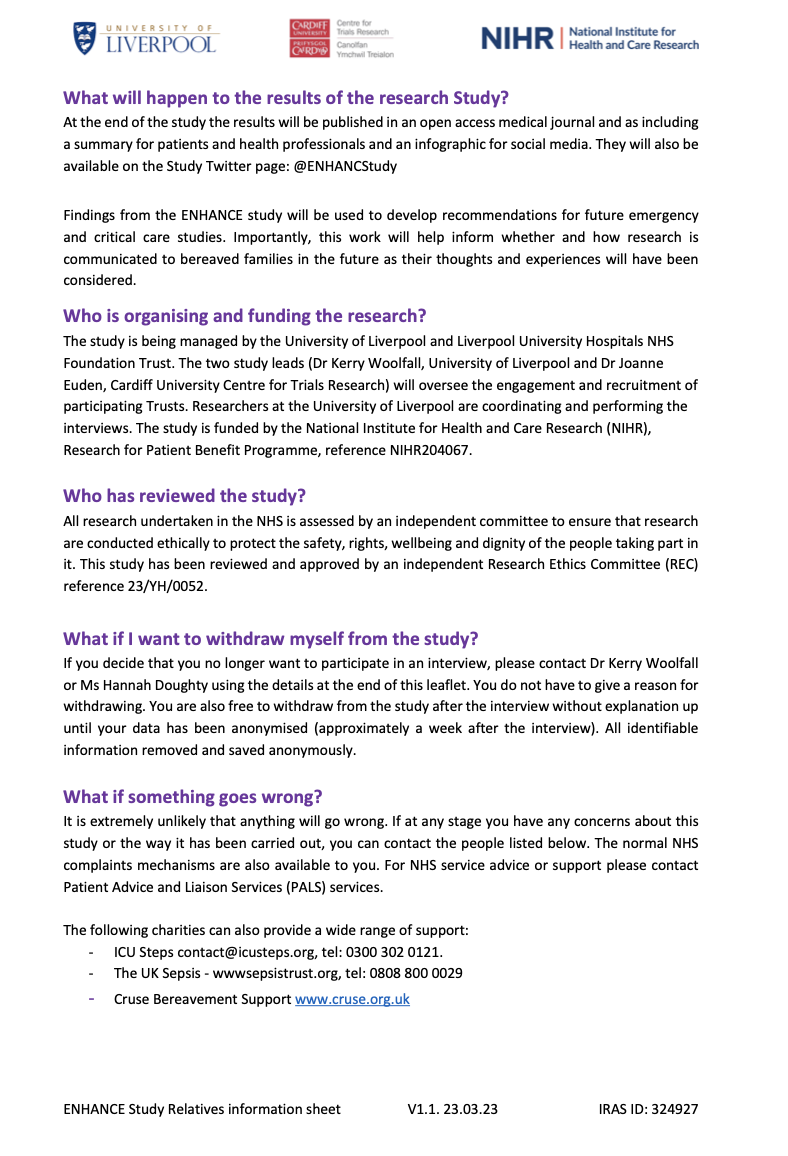
**

**
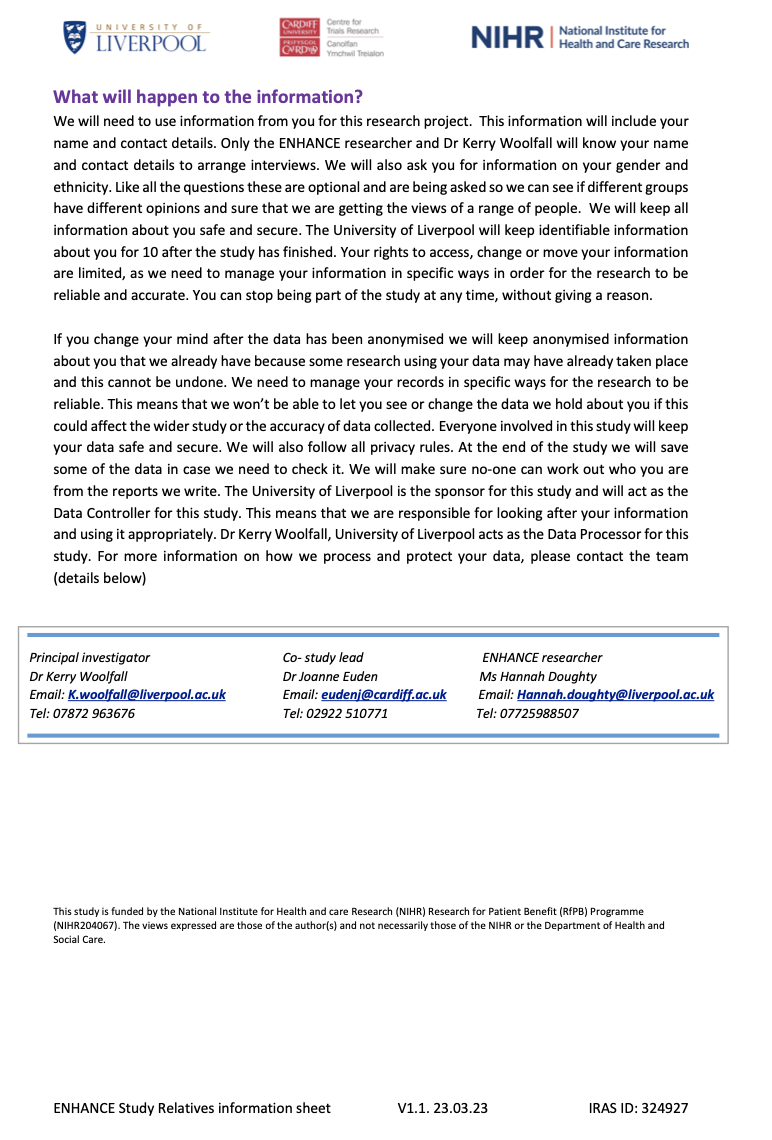
**

**
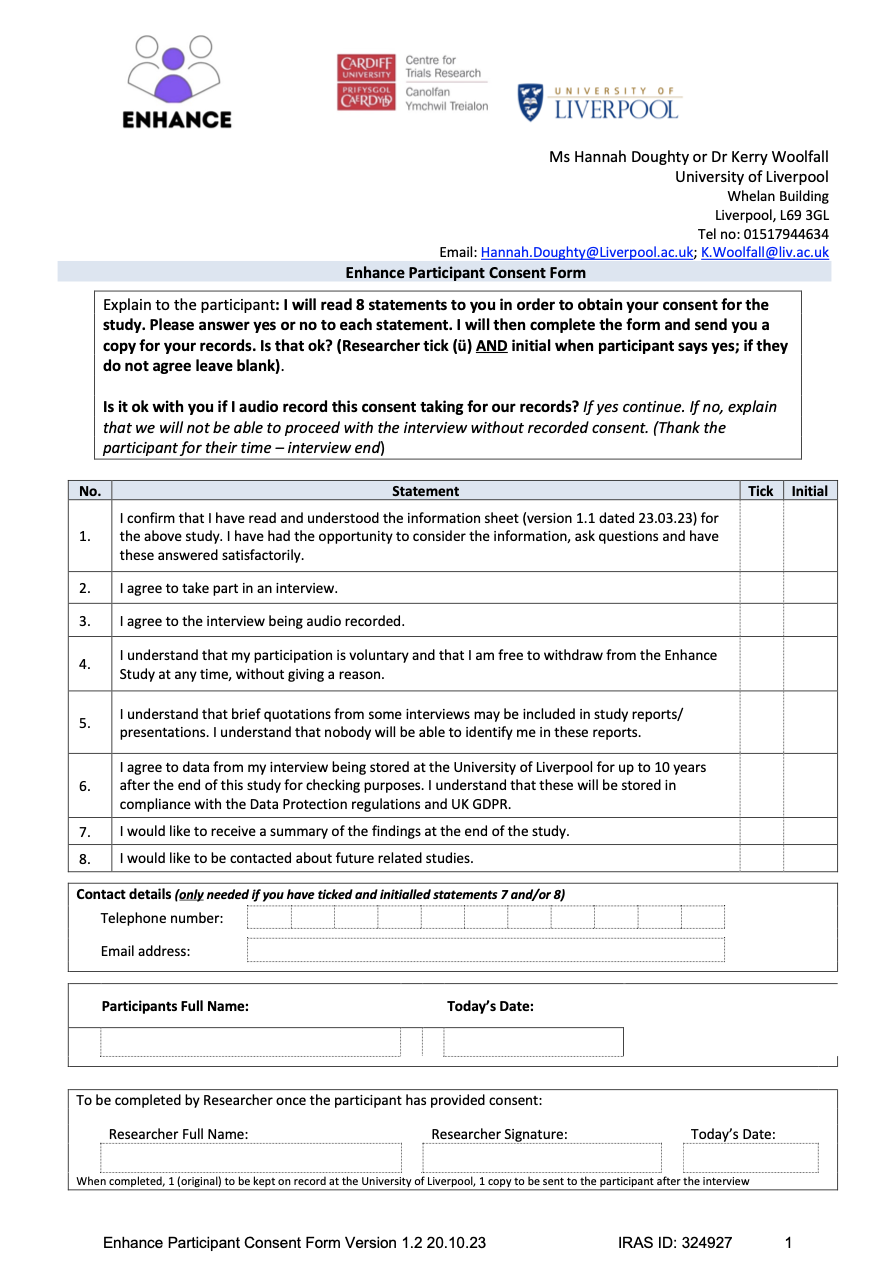
Online Resource 6.** Consent Form

**Online Resource 7.** Completed Consolidated Criteria for Reporting Qualitative research (COREQ) Checklist

| **Topic** | **Item No.** | **Guide Questions/Description** | **Reported on**  **Page No.** |
| --- | --- | --- | --- |
| **Domain 1: Research team and reﬂexivity** | | | |
| *Personal characteristics* | | | |
| Interviewer/facilitator | 1 | Which author/s conducted the interview or focus group? | 8 |
| Credentials | 2 | What were the researcher’s credentials? E.g. PhD, MD | 8 |
| Occupation | 3 | What was their occupation at the time of the study? | 8 |
| Gender | 4 | Was the researcher male or female? | 8 |
| Experience and training | 5 | What experience or training did the researcher have? | 8 |
| *Relationship with participants* | | | |
| Relationship established | 6 | Was a relationship established prior to study commencement? | N/A |
| Participant knowledge of  the interviewer | 7 | What did the participants know about the researcher? E.g. personal goals, reasons for doing the research | 8 |
| Interviewer characteristics | 8 | What characteristics were reported about the inter viewer/facilitator? E.g. Bias, assumptions, reasons and interests in the research topic | 8 |
| **Domain 2: Study design** | | | |
| *Theoretical framework* | | | |
| Methodological orientation and Theory | 9 | What methodological orientation was stated to underpin the study? E.g. grounded theory, discourse analysis, ethnography, phenomenology, content analysis | 6 |
| *Participant selection* | | | |
| Sampling | 10 | How were participants selected? E.g. purposive, convenience,  consecutive, snowball | 7 |
| Method of approach | 11 | How were participants approached? E.g. face-to-face, telephone, mail, email | 7 |
| Sample size | 12 | How many participants were in the study? | 10 |
| Non-participation | 13 | How many people refused to participate or dropped out? Reasons? | 10 |
| *Setting* | | | |
| Setting of data collection | 14 | Where was the data collected? E.g. home, clinic, workplace | 10 |
| Presence of non-  participants | 15 | Was anyone else present besides the participants and researchers? | N/A |
| Description of sample | 16 | What are the important characteristics of the sample? E.g. demographic data, date | 9 |
| Repeat interviews | 18 | Were repeat interviews carried out? If yes, how many? | N/A |
| Audio/visual recording | 19 | Did the research use audio or visual recording to collect the data? | 9 |
| Field notes | 20 | Were ﬁeld notes made during and/or after the interview or focus group? | 25 |
| Duration | 21 | What was the duration of the interviews or focus group? | 10 |
| Data saturation | 22 | Was data saturation discussed? | 9 |
| Transcripts returned | 23 | Were transcripts returned to participants for comment and/or correction? | N/A |
| **Domain 3: analysis and ﬁndings** | | | |
| *Data analysis* | | | |
| Number of data coders | 24 | How many data coders coded the data? | 24 |
| Description of the coding  tree | 25 | Did authors provide a description of the coding tree? | 24 |
| Derivation of themes | 26 | Were themes identiﬁed in advance or derived from the data? | 24 |
| Software | 27 | What software, if applicable, was used to manage the data? | 9 |
| Participant checking | 28 | Did participants provide feedback on the ﬁndings? | N/A |
| *Reporting* | | | |
| Quotations presented | 29 | Were participant quotations presented to illustrate the themes/ﬁndings? Was each quotation identiﬁed? E.g. participant number | 10 |
| Data and ﬁndings consistent | 30 | Was there consistency between the data presented and the ﬁndings? | 10 |
| Clarity of major themes | 31 | Were major themes clearly presented in the ﬁndings? | 10 |
| Clarity of minor themes | 32 | Is there a description of diverse cases or discussion of minor themes? | 10 |
