# Supplementary material for: Enhancing communication with bereaved relatives about emergency and critical care trials (ENHANCE): a mixed-methods study
Source: BMJ Open. 2026 Feb 26;16(2):e106677. doi: 10.1136/bmjopen-2025-106677 (PMC12959003; doi:10.1136/bmjopen-2025-106677)
Supplement: Supplementary file 2 [file bmjopen-2025-106677.reviewer_comments.pdf]

PEER REVIEW HISTORY

BMJ Open publishes all reviews undertaken for accepted manuscripts. Reviewers are asked to complete a checklist review form and are provided with free text boxes to elaborate on their assessment. These free text comments are reproduced below.

ARTICLE DETAILS

Title (Provisional)

Enhancing Communication with Bereaved Relatives About Emergency and Critical Care Trials (ENHANCE): A Mixed-Methods Study

Authors

Doughty, Hannah; Deja, Elizabeth; Young, Bridget; Welters, Ingeborg; Shepherd, Victoria; Milosevic, Sarah; O'Hara, Kellie; Carman, Julie; Sankar, Vinoth; Thomas-Jones, Emma; Euden, joanne; Woolfall, Kerry

---

VERSION 1 - REVIEW

**Reviewer**	**1**
**Name**	**Won Shin, Ji**
**Affiliation**	**The Ohio State University**
**Date**	**06-Aug-2025**
**COI**	**None**

Thank you for giving me an opportunity to review this paper. This paper addresses an important area of communication with bereaved families in acute care settings. However, this paper has potential methodological flaws and needs clearer descriptions for most sections. Hope you find my comments useful as you continue work on this project.

Overall, different terms were used for 'family members' all over the place, such as relatives, families, family members, and etc. The term, relative, was used for both family and patient in the introduction section, which causes confusion. Throughout the entire paper, a consistent term should be used and clearly defined.

1. My main question is: you described the study design as mixed-methods. I do not find any mixed methods component in this paper. The survey questions (Table 1) only ask about demographic information and some questions are still qualitative questions. The data analysis also involves only qualitative data analysis and the results section reports only qualitative findings. This paper should be reframed as qualitative research if there is no more quantitative component involved other than what is described here.
2. The methods section is missing a description of the study setting. It will need to include a description of standard procedures for communicating with bereaved families to show the current practice. Also, any patient clinical information hasn't

been reported or analyzed. All of this information is important to address this topic as potential factors.

3. Inclusion/exclusion criteria are not clear for bereaved relatives for surveys VS. qualitative interviews, for other participants. This is another main concern – the recruitment process is unclear. I was able to find more information in the diagram; however, a clearer description should be included in the main text.
4. If snowball sampling was used, a description of the actual method and process should be provided.
5. All the acronyms should be introduced when first used at the beginning. I suggest reviewing the paper for the accurate use of acronyms and making corrections.
6. It is good that the sampling method was designed to achieve sample diversity. However, more description is needed on how the diversity was reached, including what characteristics you considered and how it was evaluated.
7. Data analysis section should be entirely revised based on the study design. The current analysis can't be considered a mixed-methods analysis. The integration of quantitative and qualitative data is missing.
8. It says that all interviews were conducted remotely – how? What method was used? Telephone or video calls? By whom? More description is needed.

**Reviewer**	**2**
**Name**	**Castro-Avila, Ana**
**Affiliation**	**University of York, Health Sciences**
**Date**	**25-Aug-2025**
**COI**	**None**

Thanks for the opportunity to review this very important piece of work, looking into communication strategies for bereaved families when a relative dies following enrolment into an emergency or critical care study without prior consent.

This manuscript is very well-written, and I congratulate the authors on producing a very interesting read. I have minor comments that I hope will help them improve the manuscript.

Abstract:

I would like to see a bit more detail about the design. It is unclear to me whether the quantitative and qualitative components run in parallel or sequentially. If they are sequential, I would like to know which part informs which. I would also like to know how you integrated the findings from the quantitative and qualitative components. I know this is mentioned in the full-text, but it is not clear here.

The results could be structured more effectively to support your conclusion. At the moment, the conclusion states "Our findings present recommendations to support transparent, sensitive and timely communication", but the results section only refers to transparency to build trust and that family members would like to have a choice.

Methods

Lines 40-42: You might not have the answer to this, but shouldn't all trials that recruit patients without prior consent have a plan to inform the patient (or their relatives) that they were part of the trial?

Structure of the methods: I am not entirely averse to the current structure, but I did have to scroll up and down multiple times to understand the relatives' research timeline. I wonder if it would be better for the reader to have everything related to the survey first (i.e., development, eligibility, recruitment, procedure), and then, everything about the interviews. Additionally, I believe that splitting the details regarding the interviews with clinical/ research staff, ME, and relatives would be useful. It feels like the whole section "semi-structured interviews" under "procedure" addresses the interviews with relatives, but there is no clear equivalent for research staff/ME.

Figure 1 helps a lot to understand the process, but the text is not as clear as the figure.

It is not clear to me what the value of the MEO-led survey was, considering you asked very similar questions as part of the interviews. Maybe this needs further clarification.

Findings

The reporting of numbers within the findings should be consistent. Under "Early integration and format of research participation communication", you report (numerator/denominator; percentage). However, on page 11, one number is reported as "numerator; percentage", while another one is reported as a percentage only.

Discussion

Page 14, lines 28-53: This paragraph essentially repeats the findings. I think you could express the same ideas more succinctly.

VERSION 1 - AUTHOR RESPONSE

Reviewer 1's Comments:

- Overall, different terms were used for 'family members' all over the place, such as relatives, families, family members, and etc. The term, relative, was used for both family and patient in the introduction section, which causes confusion. Throughout the entire paper, a consistent term should be used and clearly defined.
- We thank reviewer 1 for their valuable feedback. In response, we have revised the manuscript to consistently use the term *"relatives"* when referring to the bereaved, and *"family members"* when referring to the deceased patient. This ensures clarity and consistency in our terminology, enhancing the readability of the paper and preventing any potential confusion.
- My main question is: you described the study design as mixed-methods. I do not find any mixed methods component in this paper. The survey questions (Table 1) only ask about demographic information and some questions are still qualitative questions. The data analysis also involves only qualitative data analysis and the results section reports only qualitative findings. This paper should be reframed as qualitative research if there is no more quantitative component involved other than what is described here.
- The MEO-led survey used a quantitative design with binary, closed-ended questions, producing numerical rather than qualitative data. These responses were analysed descriptively, as outlined in the data analysis section of the manuscript, and presented as quantitative data alongside the qualitative findings in the results section. We have ensured

this distinction is clearly explained in the data analysis section (pages 9-10) and in the results (from page 12 onwards), where the numerical findings are presented more explicitly. We have also clarified, at the end of the data analysis section, how we synthesised the quantitative and qualitative data.

- The methods section is missing a description of the study setting. It will need to include a description of standard procedures for communicating with bereaved families to show the current practice. Also, any patient clinical information hasn't been reported or analyzed. All of this information is important to address this topic as potential factors.
  We have added the study setting to the methods section (page 6). Currently, there is no standard procedure or legal requirement for communicating with bereaved relatives about their deceased family member's participation in research, and most emergency or critical care studies do not include a process for notifying bereaved relatives. This has been made clearer in the introduction on page 5. As our focus was to understand bereaved relatives' views and use these to inform practice and recommendations, we did not collect patient clinical information, as this was outside the scope of our research and not required to address our aim and objectives.
- Inclusion/exclusion criteria are not clear for bereaved relatives for surveys VS. qualitative interviews, for other participants. This is another main concern – the recruitment process is unclear. I was able to find more information in the diagram; however, a clearer description should be included in the main text.
  We have revised the layout so that all information related to the MEO-led survey (including eligibility, recruitment process, and procedure) are presented consecutively, followed by the same structure for the semi-structured interviews. We hope this reorganisation makes the description of the methodology for both components clearer and easier to follow.
- If snowball sampling was used, a description of the actual method and process should be provided.
  We have added a sentence related to snowball sampling on page 9 of the manuscript.
- All the acronyms should be introduced when first used at the beginning. I suggest reviewing the paper for the accurate use of acronyms and making corrections.
  All acronyms used have been reviewed throughout the manuscript.
- It is good that the sampling method was designed to achieve sample diversity. However, more description is needed on how the diversity was reached, including what characteristics you considered and how it was evaluated.
  We have revised the manuscript to include details about the participant characteristics and the approach used to evaluate them in order to achieve sample diversity. These additions can be found on page 9 of the manuscript.
- Data analysis section should be entirely revised based on the study design. The current analysis can't be considered a mixed-methods analysis. The integration of quantitative and qualitative data is missing.
  As noted in our response to point 2, the data analysis section (pages 9-10) have been revised to clarify that quantitative and qualitative data were integrated and synthesised using constant comparison, informed by grounded theory.
- It says that all interviews were conducted remotely – how? What method was used? Telephone or video calls? By whom? More description is needed.
  We have revised the manuscript to provide further detail about how the remote interviews were conducted. Information on who conducted the interviews was included in the opening sentence of the "Procedure" section (page 9). Additional details regarding the number of interviews conducted by telephone versus Microsoft Teams/Zoom have been added on page 11.

<u>Reviewer 2's Comments:</u>

- <u>Abstract:</u>
  I would like to see a bit more detail about the design. It is unclear to me whether the quantitative and qualitative components run in parallel or sequentially. If they are sequential, I would like to know which part informs which. I would also like to know how you integrated

the findings from the quantitative and qualitative components. I know this is mentioned in the full-text, but it is not clear here.

- We thank reviewer 2 for their valuable feedback. We have added a sentence on page 3 clarifying that the qualitative and quantitative components were conducted simultaneously and we have explained how the findings were integrated.
- The results could be structured more effectively to support your conclusion. At the moment, the conclusion states "Our findings present recommendations to support transparent, sensitive and timely communication", but the results section only refers to transparency to build trust and that family members would like to have a choice.
- We have revised the results section so that the results are presented in a way that more clearly supports our concluding statements. Please see page 3.
- Methods
  Lines 40-42: You might not have the answer to this, but shouldn't all trials that recruit patients without prior consent have a plan to inform the patient (or their relatives) that they were part of the trial?
- Currently, there is no standard procedure or legal requirement for communicating with bereaved relatives about their deceased family member's participation in research, and most emergency or critical care studies do not include a process for notifying bereaved relatives. This has been made clearer in the introduction on page 5.
- Structure of the methods: I am not entirely averse to the current structure, but I did have to scroll up and down multiple times to understand the relatives' research timeline. I wonder if it would be better for the reader to have everything related to the survey first (i.e., development, eligibility, recruitment, procedure), and then, everything about the interviews.
- We have revised the layout so that all information related to the MEO-led survey (including eligibility, recruitment process, and procedure) are presented consecutively, followed by the same structure for the semi-structured interviews. Please see page 6.
- Additionally, I believe that splitting the details regarding the interviews with clinical/ research staff, ME, and relatives would be useful. It feels like the whole section "semi-structured interviews" under "procedure" addresses the interviews with relatives, but there is no clear equivalent for research staff/ME.
- The procedures for the staff and relatives' interviews have been separated and described more clearly within the methods section (page 9).
- Figure 1 helps a lot to understand the process, but the text is not as clear as the figure. It is not clear to me what the value of the MEO-led survey was, considering you asked very similar questions as part of the interviews. Maybe this needs further clarification.
- We have revised the text on page 7 to make the purpose of the MEO-led survey clearer.
- Findings
  The reporting of numbers within the findings should be consistent. Under "Early integration and format of research participation communication", you report (numerator/denominator; percentage). However, on page 11, one number is reported as "numerator; percentage", while another one is reported as a percentage only.
- We have reviewed the manuscript to ensure that all quantitative figures are reported consistently, including numerator, denominator, and percentage.
- Discussion
  Page 14, lines 28-53: This paragraph essentially repeats the findings. I think you could express the same ideas more succinctly.
- We had followed the BMJ Open author guidance to present the main findings at the start of the discussion, we have now reworded this paragraph to present the key messages more succinctly (page 14).

VERSION 2 - REVIEW

Reviewer 2

Name Castro-Avila, Ana
Affiliation University of York, Health Sciences
Date 13-Oct-2025
COI

Thanks for the opportunity to review the revised version of this manuscript exploring potential communication strategies for informing bereaved relatives when a patient has died following enrolment into an emergency or critical care study without prior consent.

I have very minor comments:

Abstract:

In "design and setting", you say that you conducted interviews with bereaved relatives and research and clinical staff across the United Kingdom, and medical examiner/medical examiner officers based in England and Wales. However, in "participants" you refer to relatives, medical examiners/ officers, research nurses, doctors, and bereavement nurses. For consistency, it is better to use the same language (i.e., research and clinical staff= research nurses, doctors and bereavement nurses).

Methods

Page 10, lines 3-14: this could be improved. You list all the inclusion criteria for the three different groups of participants, but it is not fully clear which one applies to which group. Also, I was not expecting criterion 2, and I wonder how it would affect the results if you had relatives of patients who died suddenly but were not part of a research without prior consent trial.

Under procedure, it is still not clear which parts are applicable to patients, which ones to research and clinical staff, and which ones to medical examiners.

Results

Page 14, lines 9-13: these appear underlined in my version, and I can only assume it is an error

Page 14, from line 33 up to page 15, line 35: this could be shortened substantially if you highlight the main demographic characteristics and refer the reader to the tables for the rest of the information.

VERSION 2 - AUTHOR RESPONSE

Reviewer 2's Comments:

Abstract:
In "design and setting", you say that you conducted interviews with bereaved relatives and research

and clinical staff across the United Kingdom, and medical examiner/medical examiner officers based in England and Wales. However, in "participants" you refer to relatives, medical examiners/ officers, research nurses, doctors, and bereavement nurses. For consistency, it is better to use the same language (i.e., research and clinical staff= research nurses, doctors and bereavement nurses).
We thank reviewer 2 for their valuable feedback. We have revised the manuscript to use the term research and clinical staff throughout for clarity and consistency.

Methods
Page 10, lines 3-14: this could be improved. You list all the inclusion criteria for the three different groups of participants, but it is not fully clear which one applies to which group. Also, I was not expecting criterion 2, and I wonder how it would affect the results if you had relatives of patients who died suddenly but were not part of a research without prior consent trial.
Tracked changes have been made to this section in the manuscript, to enhance the clarity of the eligibility criteria.

Under procedure, it is still not clear which parts are applicable to patients, which ones to research and clinical staff, and which ones to medical examiners.
Tracked changes have been made to this section of the manuscript to enhance the clarity of the procedure.

Results
Page 14, lines 9-13: these appear underlined in my version, and I can only assume it is an error
The current file has no underlining for this section.

Page 14, from line 33 up to page 15, line 35: this could be shortened substantially if you highlight the main demographic characteristics and refer the reader to the tables for the rest of the information.
Tracked changes have been made to the results section to enhance the clarity of the demographic information. The characteristics of participating relatives are now summarised in Table 3, and the reader is directed there for full details.
